# Ocrelizumab in Patients with Active Primary Progressive Multiple Sclerosis: Clinical Outcomes and Immune Markers of Treatment Response

**DOI:** 10.3390/cells11121959

**Published:** 2022-06-17

**Authors:** Marina Boziki, Christos Bakirtzis, Styliani-Aggeliki Sintila, Evangelia Kesidou, Evdoxia Gounari, Aliki Ioakimidou, Vasiliki Tsavdaridou, Lemonia Skoura, Asimina Fylaktou, Vasiliki Nikolaidou, Maria Stangou, Ioannis Nikolaidis, Virginia Giantzi, Eleni Karafoulidou, Paschalis Theotokis, Nikolaos Grigoriadis

**Affiliations:** 1 Multiple Sclerosis Center of the 2nd Neurological University Department, School of Medicine, Aristotle University of Thessaloniki, AHEPA General University Hospital, 54636 Thessaloniki, Greece; bozikim@auth.gr (M.B.); bakirtzischristos@yahoo.gr (C.B.); lina17puma@yahoo.gr (S.-A.S.); bioevangelia@yahoo.gr (E.K.); ioannisnikol78@gmail.com (I.N.); virginiagiantzi@gmail.com (V.G.); elenikarafoulidou95@hotmail.com (E.K.); ptheotokis@auth.gr (P.T.); 2 Microbiology Laboratory, Department of Immunology, AHEPA University Hospital, 54636 Thessaloniki, Greece; evigounari@yahoo.com (E.G.); aliki_ioakimidou@yahoo.gr (A.I.); vaso.tsavdaridou@gmail.com (V.T.); lemskour@auth.gr (L.S.); 3 National Peripheral Histocompatibility Center, Immunology Department, Hippokration General Hospital, 54642 Thessaloniki, Greece; fylaktoumina@gmail.com (A.F.); basoniko@hotmail.com (V.N.); 4 Department of Nephrology, Medical School, Aristotle University of Thessaloniki, Hippokration Hospital, 54642 Thessaloniki, Greece; mstangou@auth.gr; 5 Special Unit for Biomedical Research and Education, School of Medicine, Aristotle University of Thessaloniki, 54636 Thessaloniki, Greece

**Keywords:** primary progressive multiple sclerosis, disease activity, ocrelizumab, disease-modifying treatment, depletion treatment, treatment response, immunophenotype, MRI volumetry, cognition, cytokine profile

## Abstract

Ocrelizumab is a B-cell-depleting monoclonal antibody approved for the treatment of relapsing-remitting multiple sclerosis (RRMS) and active primary progressive MS (aPPMS). This prospective, uncontrolled, open-label, observational study aimed to assess the efficacy of ocrelizumab in patients with aPPMS and to dissect the clinical, radiological and laboratory attributes of treatment response. In total, 22 patients with aPPMS followed for 24 months were included. The primary efficacy outcome was the proportion of patients with optimal response at 24 months, defined as patients free of relapses, free of confirmed disability accumulation (CDA) and free of T1 Gd-enhancing lesions and new/enlarging T2 lesions on the brain and cervical MRI. In total, 14 (63.6%) patients and 13 patients (59.1%) were classified as responders at 12 and 24 months, respectively. Time exhibited a significant effect on mean absolute and normalized gray matter cerebellar volume (F = 4.342, *p* = 0.23 and F = 4.279, *p* = 0.024, respectively). Responders at 24 months exhibited reduced peripheral blood ((%) of CD19+ cells) plasmablasts compared to non-responders at the 6-month point estimate (7.69 ± 4.4 vs. 22.66 ± 7.19, respectively, *p* = 0.043). Response to ocrelizumab was linked to lower total and gray matter cerebellar volume loss over time. Reduced plasmablast depletion was linked for the first time to sub-optimal response to ocrelizumab in aPPMS.

## 1. Introduction

Multiple sclerosis (MS) is an inflammatory and neurodegenerative disease of the central nervous system, with T-cells of the adaptive immune system being regarded as the main mediators of the disease [1]. Recently, a role for B-cells in relation to disease activity and progression has emerged [2]. The contribution of B-cells in MS pathogenesis has long been suspected; however, several mechanisms of this contribution have recently been elucidated. For instance, oligoclonal bands (OCBs) have long been utilized as diagnostic markers for MS, in the frame of the widely diagnostic criteria [3]. OCBs are antibodies of the IgG class that are produced intrathecally in the context of the neuroinflammatory process in the cerebrospinal fluid (CSF) of people with MS. Clonotypic analysis indicates that OCBs are produced by plasmablasts and/or plasma cells that infiltrate the CNS from the periphery [4,5]. However, in addition to their role in antibody production, B-cells are likely to exert an effect on T-cells in the context of MS evolution. Specifically, B-cells are known to act as antigen-presenting cells, bearing molecules of the major histocompatibility complex (MHC) class II [6] and thus being capable of antigen internalization and subsequent participation in the APC-T-cell immunological synapse. B-cells were also shown to affect T-cell effector properties via the production of pro-inflammatory cytokines and further induction of pro-inflammatory effector phenotype in T-cells, thus contributing to MS pathogenesis [7]. Moreover, the identification of germinal center-like follicles in the leptomeninges of patients with secondary progressive MS (SPMS) shed new light on the concept of intrathecal B-cell maturation and its role in sustaining chronic inflammation in the progressive forms of MS [8]. Clonotypic homology across different counterparts, namely, blood, CSF and the CNS lesions, further supports the functional relevance of these clonotypes to pathogenic processes [9,10].

In MS, neuroinflammatory and neurodegenerative processes lie over a continuum, with the relative contribution of neurodegeneration increasing in the progressive forms of the disease [11]. We and others have previously described cognitive impairment, affect alterations and behavioral disturbance with deficient inhibitory control over the course of the disease [12,13,14]. Moreover, recent evidence indicates that the integration patterns of emotional stimuli into core executive functions, such as inhibitory control, may influence behavior in several neurodegenerative and psychiatric diseases [15]. The involvement of cortical and also subcortical areas, such as the amygdala, may, in fact, alter emotional perception, thus interfering with social cognition and functioning [16]. More specifically, the prefrontal cortex and the amygdala have been recognized to be among the anatomical brain regions implicated in human emotional learning and functional circuits, known to be impaired in MS and other neurodegenerative diseases [17]. The prefrontal cortex, a major cognitive structure, has been recognized to influence the activity of subcortical areas, such as the amygdala and hippocampus, thus providing a neurophysiological basis for neuro-cognitive emotional regulation [18]. Brain volume loss and the associated disturbance of neuronal circuits has been described in MS and other neurodegenerative diseases, and extensive research implicating precision diagnostics and machine-learning approaches in the management of the disease are currently under development [19]. These applications collectively aim to integrate genetic, clinical, radiological and molecular data in order to fingerprint the mechanisms of disease evolution and to identify diagnostic and/or prognostic biomarkers in relation to neuroinflammatory and/or neurodegenerative disease [20,21]. Vitamin D [22], molecular markers of oxidative stress [23] and markers indicative of systemic and cellular metabolic dysregulation [24,25,26], such as the tryptophan–kynurenine pathway, a metabolic pathway impaired in a wide range of neurodegenerative and autoimmune diseases, also implicated in the gut–brain axis homeostasis [27], are the object of extensive research.

Ocrelizumab is a B-cell-depleting anti-CD20 monoclonal antibody approved for the treatment of RRMS, as well as for active primary progressive MS (PPMS) [28]. In patients with relapsing MS, ocrelizumab was associated with improved outcomes of clinical and radiological activity, as well as with reduced rates of confirmed disability progression compared to patients receiving interferon β-1a [29]. In addition, in patients with PPMS, ocrelizumab was associated with lower rates of clinical and MRI progression, as compared with placebo [30]. Ocrelizumab targets primarily naïve and memory B-cells, whereas CD20-negative plasma cells escape the depletion, thus leaving the antibody production essentially unaffected. Upon repopulation, naïve and immature B-cells mainly re-emerge, together with a few memory B-cells and plasmablasts [31]. Evidence stemming from studies on ocrelizumab, as well as on previously available B-cell-depleting agents, such as rituximab, indicates that anti-CD20 targeting B-cell depletion also leads to elimination of a small population of CD20-bearing T-cells (CD20^dim^ T-cells) and to significant alterations in the T-cell effector phenotype and the cytokine production by T-cells [32,33,34]. In the clinical setting, however, the optimal treatment scheme, as well as predictors of poor response to anti-CD20 treatment for MS and other immune-mediated neurological diseases, remain to be defined. B-cell reconstitution profile may provide valuable information with respect to the optimal treatment response, but the available studies are limited [35,36,37,38,39,40,41].

The present study aims to assess the efficacy of ocrelizumab in a cohort of patients with active progressive MS and to dissect the clinical, radiological and laboratory attributes of treatment response.

## 2. Materials and Methods

### 2.1. Patients

Twenty-two patients with active primary progressive multiple sclerosis (PPMS) were included in this prospective, uncontrolled, open-label, observational study. All patients were followed by the Multiple Sclerosis Center of the 2nd Department of Neurology of the Aristotle University of Thessaloniki in AHEPA University General Hospital. Patients received ocrelizumab from February 2018 until August 2021, according to the European Medicines Agency (EMA) label. Upon ocrelizumab treatment initiation, all patients were over 18 years old. Patients who previously received immunosuppressants were excluded from the study. A minimum treatment duration with ocrelizumab for 24 months was necessary for inclusion in the analysis. Treatment with ocrelizumab was administered initially as two paired doses of 300 mg i.v. separated by a two-week interval, and cycles were repeated as single infusions of 600 mg i.v. every 6 months. The study was conducted in accordance with the Helsinki Declaration. All participants provided written informed consent. The study received the approval of the Bioethics’ Committee of the School of Medicine of the Aristotle University of Thessaloniki (7/20.04.2021-7.382). Power analysis was conducted by the use of G*Power 3.1.9.7 for Windows. Analysis parameters for ANOVA—repeated measures, within–between interaction—were set as follows: effect size f = 0.25; α error probability = 0.05; power (1–β error probability) = 0.8; number of groups = 2, number of measurements = 4. Total sample size was calculated as N = 24 with actual power 0.82.

### 2.2. Study Procedures

Clinical and demographic characteristics of each patient were collected. Participants underwent neurological examination through the expanded disability status scale (EDSS) [42,43] by a certified EDSS rater. Cognitive assessment was performed with the Greek validated version of the Brief International Cognitive Assessment of Multiple Sclerosis (BICAMS) [12,44,45]. This battery included the Symbol Digit Modalities Test (SDMT) for the assessment of information processing speed [46], the Greek Verbal Learning Test (GVLT) [47,48] and the Brief Visuospatial Memory Test-Revised (BVMT-R) [49] for the assessment of verbal and visuospatial memory, respectively. The modified fatigue impact scale (MFIS) [50,51] was administered in order to quantify self-reported fatigue. All tests and measures were administered in the same order to all participants in a quiet room without distractions. The cognitive assessment was conducted at baseline and at 6-month intervals thereafter, using alternate forms. For assessment in relation to MRI measurements, only the time points corresponding to MRI assessment were taken into account, therefore, at baseline, at 12 and at 24 months.

MRI evaluation was performed prior to treatment onset (baseline) and annually thereafter. MRI studies were conducted in various facilities due to the fact that they were conducted as part of routine clinical practice, but they were performed with the same imaging protocol, in a 1.5T scanner. All patients received gadolinium at baseline, whereas gadolinium was not administered at follow-up MRI, as per recent MRI guidelines for the monitoring of people with MS [52]; therefore, the number of enhancing T1 lesions was not applicable as a measure in this study. Three-dimensional T1-weighted and T2-weighted FLAIR images with 1mm slice thickness were used in order to perform the analyses. First, all scans were evaluated for moving artifacts; then, we used the VolBrain^TM^ platform [53] for the volumetric and lesion load analysis, as in Refs [54,55,56]. Lesion analysis with VolBrain^TM^ was not conducted for cervical MRI, as this analysis is not available. However, cervical new T2 lesions evidenced by routine MRI evaluation were taken into account for the overall assessment of treatment response at 12 and at 24 months.

### 2.3. B-Cell Immunophenotype

**Figure 1:**
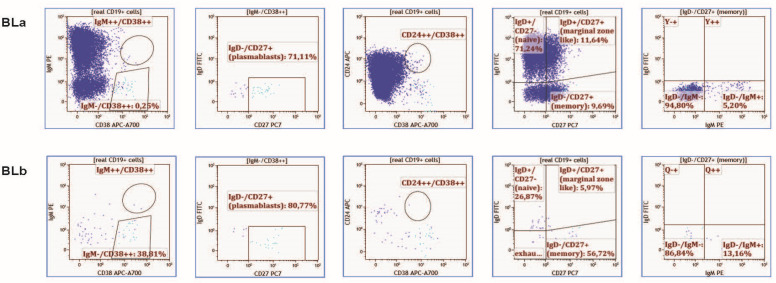
B-cell immunophenotype gating example. Representative consecutive FACS plots showing B-cell sub-populations at baseline pre-first infusion (BLa) and 15 days after treatment with the first infusion and pre-second infusion (BLb).

B-cell populations were assessed by a highly sensitive 9-colour multiparametric flow cytometry (FC) analysis of peripheral blood (PB). As ocrelizumab is administered at treatment initiation as two equally divided doses, 15 days apart, and as a single infusion at 6-month intervals thereafter, baseline analysis included two time points. More specifically, analysis was performed immediately before treatment infusion, at pre-first baseline infusion (BLa), as well as 15 days afterward, at pre-second baseline infusion (BLb). The analysis was repeated in 6-month intervals thereafter, again, pre-infusion. Primary B-cell subsets were defined as follows: plasmablasts (CD19+(weak)/CD27++/CD38++), transitional B-cells (CD19+/CD38++/IgM++/24++), marginal-zone-like B-cells (CD19+/CD27+/IgD+), class-switched memory B-cells (CD19+/CD27+/IgM-/IgD-), non-switched CD27+ memory B-cells (CD19+/CD27+/IgM+/IgD−), naïve B-cells (CD19+/CD27-/IgD+). CD14+ and CD3+ cells were selectively gated out as contaminating myeloid and T lymphocytes in order to achieve a more accurate enumeration of B-cells and, subsequently, B-cell subpopulation immunophenotyping. For B-cell immunophenotype, samples were stained with IgD-FITC and IgM-PE Polyclonal Anti-Human Rabbit (Fab2) antibodies, as well as CD19-ECD clone J3-119, CD27-PC7 clone 1A4LDG5, CD24-APC clone ALB9, CD38-APC-A700 clone LS198-4-3, CD14-APC-A750 clone RMO52, CD3-PB clone UCHT1 and CD45-KrOr clone J33 antibodies, after bulk lysis. Samples were processed on a Navios cytometer (Beckman-Coulter) and analyzed with KALUZA software. Immunophenotype strategy was applied as in Refs [57,58]. A gating example of BLa and BLb time points is presented in Figure 1.

### 2.4. Serum Cytokine Profile

Serum cytokine analysis (IL-6; INFγ; TNFα; IL-1β; IL-12; IL-2; IL-4; IL-5; IL-10; IL-17A) was performed with flow cytometry, with the use of a multiplexed bead-based immunoassay panel (AimPlex Biosciences, Inc., Pomona, CA, USA) [59,60]. Samples were acquired in a NAVIOS Beckman Coulter Three Laser 10-color cytometer and analyzed with FlowJo™ Software (BD Biosciences, Becton, Dickinson and Company, NJ, USA).

### 2.5. Clinical, MRI and Laboratory Outcomes

#### 2.5.1. Primary Outcome

The primary efficacy outcome was the proportion of patients with optimal response at 24 months, hereafter mentioned as “responders”. Optimal response was defined as the proportion of patients free of relapse, free of confirmed disability accumulation (CDA) evidenced by EDSS evaluation and free of new and/or enlarging T2 lesions on the brain and cervical MRI. Of note, all patients during the study remained free of relapse in the context of PPMS. In this respect, CDA occurred in the absence of relapses, thus essentially corresponding to confirmed disability progression (CDP) by EDSS evaluation [61]. Although relapses are not typical for patients with PPMS, nevertheless, as per the 2013 revised classification of the types of MS [62], a patient with progressive disease, including primary progressive disease, may present as “active with progression” or “active without progression”, whereby activity is determined by clinical relapses assessed at least annually, and/or MRI activity is typically not completely excluded. Due to this fact, we retained the assessment of possible relapses in the definition of treatment response in the present study. Patients with evidence of CDA and/ and/or new/enlarging T2 lesions on the brain and cervical MRI are hereafter collectively mentioned as “non-responders”.

#### 2.5.2. Secondary Outcomes

- The proportion of patients with optimal response at 12 months. Optimal response is defined as absence of relapses, absence of new/enlarged T2 lesions on the brain and cervical MRI and absence of CDA, defined as 1 point of EDSS increase (0.5 point if baseline EDSS ≥ 5.5, confirmed after 6 months from the previous last evaluation) at 12 and at 24 months.
- The mean time to optimal response for patients who exhibited optimal response at 24 months. As per study design, patients were evaluated every 6 months.
- The proportion of patients with CDA defined as 1 point of EDSS increase (0.5 point if baseline EDSS ≥ 5.5), at 12 and at 24 months, relative to the last previous evaluation time point.
- The proportion of patients with MRI activity at 12 and 24 months (defined as the presence of new/enlarged T2 lesions with respect to previous brain MRI.

#### 2.5.3. Exploratory Outcomes

- Mean change in EDSS from baseline at 12- and 24-month point estimates (for all patients and for responders vs. non-responders at 24 months).
- Mean change in total number of new and/or enlarged T2 lesions at 12- and 24-month point estimates (for all patients and for responders vs. non-responders at 24 months).
- Mean change in cognitive performance scales’ scores, namely, BICAMS (including SDMT, GVLT, BVMT-R), for the assessment of processing speed, verbal and visuospatial memory, respectively, from baseline at 12- and 24-month point estimates (for all patients and for responders vs. non-responders at 24 months).
- Mean change in volumetric parameters in cm^3^ and in percentage of total brain volume from baseline at 12- and 24-month point estimates (for all patients and for responders vs. non-responders at 24 months). Example measurements include, but are not restricted to, overall, cerebrum and cerebellar white and gray matter, brainstem and areas of subcortical gray matter.
- Mean change in lesion (white matter) analysis parameters, namely, lesion count, lesion volume in cm^3^ and normalized lesion volume, as well as overall lesion burden, from baseline at 12- and 24-month point estimates (for all patients and for responders vs. non-responders at 24 months).
- Possible association between mean EDSS score and/or mean cognitive scores and MRI volumetry and/or lesion analysis at 12- and 24-month point estimates.
- Mean change in B-cell subtypes from baseline at 6-, 12-, 18- and 24-month point estimates (for all patients and for responders vs. non-responders at 24 months).
- Mean change in serum cytokine profile from baseline at 6- and 12-month point estimates (for all patients and for responders vs. non-responders at 24 months).

### 2.6. Statistical Analysis

Normality was assessed by use of the Kolmogorov–Smirnoff test. At point estimates, the non-parametric Mann–Whitney U-test and Chi-square test were used for the comparison of continuous variables (and the mean change from baseline for continuous variables) and dichotomous/categorical variables, respectively. For evaluation of the effect of time on mean EDSS, the mean number of new/enlarged T2 lesions and mean scores for cognitive tests at 12 and at 24 months, as well as MRI volumetry and lesion analysis parameters compared to baseline, general linear models in a repeated-measures setting were applied, according to which, gender, age (years), MS duration (years), EDSS at baseline and degree of brain MRI activity at baseline (number of new/enlarging T2 and Gd+ lesions) were used as covariates, and group allocation based on the response at 24 months was used as a between-subjects factor. Similarly, for the comparison of mean change from baseline at 12- and 24-month point estimates between responders and non-responders, a repeated-measures general linear model was implemented.

Possible association between mean EDSS score and/or mean cognitive scores and volumetry and/or lesion analysis were evaluated with the use of hierarchical linear models (HLM) with repeated-measures data. For this reason, the mixed procedure was implemented, with each set of repeated measures of EDSS and/or cognitive score defined as a dependent variable, case ID defined as a factor, and time, as well as each set of the respective repeated measures of volumetry or lesion analysis, defined as covariates. Covariates were set as fixed effects, whereas time was set as a random effect. For each model, the −2 restricted log likelihood, as well as the estimates and the significance of fixed effects were assessed. The analysis was conducted with the use of SPSS 27.0 (IBM Corp, Armonk, NY, USA). A significance level of 0.05 was taken into account.

## 3. Results

### 3.1. Baseline Characteristics, Safety and Treatment Withdrawal

Patients’ baseline characteristics are presented in Table 1.

In total, 12 male and 10 female patients with PPMS were included in the study, of mean age 48.5 ± 1.69 years. Mean disease duration was 9.05 ± 0.92 years, whereas time from diagnosis was 5.59 ± 0.84 years. Mean EDSS was 4.91 ± 0.3. Overall, six patients exhibited cardiovascular comorbidities, whereas nine patients exhibited other (non-cardiovascular) comorbidities, with affective disorder being the most prevalent. Retrospectively, responders at 24 months vs. non-responders did not differ in terms of baseline characteristics.

Seven patients presented with adverse events during ocrelizumab treatment. L5 radiculopathy, meningioma, urinary tract infection, deep venous thrombosis, cholecystitis (surgery-laparoscopic cholecystectomy) and migraine crisis presented in separate patients. These AEs were resolved and were not linked to ocrelizumab discontinuation. In addition, two patients, one concurrently with meningioma and one concurrently with cholecystitis, presented with serious dermatitis. Dermatitis in these two patients led to ocrelizumab discontinuation; however, the AE occurred at the end of the follow-up period of 24 months.

### 3.2. Primary and Secondary Outcomes

Primary and secondary outcomes are presented in Table 2.

In total, 14 (63.6%) patients were classified as responders at 12 months, whereas 13 patients (59.1%) were classified as responders at 24 months. At 12 months, 14 (63.6%) patients remained free of CDA, and 20 (90.9%) patients were free of new/enlarged T2 lesions on the brain and cervical MRI. At 24 months, 13 patients (59.1%) remained free of CDA, whereas 20 (90.9%) patients were free of new/enlarged T2 lesions on the brain and cervical MRI. The mean time to optimal response (calculated for responders at 24 months) was 8.73 ± 1.41 months.

### 3.3. Exploratory Outcomes

**Figure 2:**
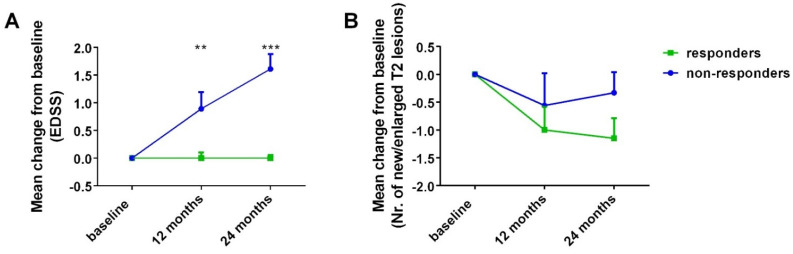
Mean change in EDSS (**A**) and new/enlarged T2 lesions (**B**) on the brain and cervical MRI at 12 and at 24 months from baseline for responders at 24 months vs. non-responders at 24 months. EDSS: Expended Disability Status Scale; NEDA: No Evidence of Disease Activity. Bars represent mean values ± standard error of mean. ** *p* < 0.01; *** *p* < 0.001.

For all patients, mean EDSS was 5.27 ± 0.28 and 5.57 ± 0.28 at 12 and at 24 months, respectively. For responders at 24 months, mean EDSS was 5.15 ± 0.36 and 5.15 ± 0.35 at 12 and at 24 months, respectively. For non-responders at 24 months, mean EDSS was 5.44 ± 0.47 and 6.17 ± 0.41 at 12 and at 24 months, respectively. Mean EDSS did not differ at point estimates between the two groups, although a tendency toward a difference at 24 months was evident (*p* = 0.695 and *p* = 0.096 for 12 and 24 months, respectively). However, mean EDSS change from baseline was different between the two groups at the 12-month point estimate (mean EDSS change of 0.00 ± 0.1 for responders at 24 months vs. 0.89 ± 0.3 for non-responders at 24 months, *p* = 0.003), as well as the 24-month point estimate (mean EDSS change of 0.00 ± 0.06 for responders at 24 months vs. 1.61 ± 0.27 for non-responders at 24 months, *p* < 0.001). The mean EDSS change between 12 and 24 months was 0.00 ± 0.06 for responders at 24 months vs. 0.72 ± 0.09 for non-responders at 24 months (*p* < 0.001) (Figure 2A). Overall, time did not have a significant effect on EDSS, evaluated with a general linear model in a repeated-measures setting (F = 0.821, *p* = 0.399). However, an effect of time on EDSS was evident when group allocation based on response at 24 months was taken into account in the model as a between-subjects factor (F = 3.922, *p* = 0.041), and the response-at-24-months group allocation exhibited a prominent, significant effect of EDSS change over time (F = 27.268, *p* < 0.001).

At baseline, EDSS was significantly correlated with normalized brainstem volume (Pearson’s r: −0.466, *p* = 0.038), with absolute (cm^3^) and with normalized thalamus volume (Pearson’s r: −0.524, *p* = 0.018 and Pearson’s r: −0.534, *p* = 0.015, respectively) and exhibited a tendency to correlate with the overall white matter lesion burden (Pearson’s r: 0.382, *p* = 0.096). Similarly, fatigue was correlated at baseline with absolute (cm^3^) and with normalized thalamus volume (Pearson’s r: −0.435, *p* = 0.055 and Pearson’s r: −0.541, *p* = 0.014, respectively), as well as with normalized cerebellum volume (Pearson’s r: −0.507, *p* = 0.023) and exhibited a tendency to correlate with absolute and with normalized lesion volume (Pearson’s r: 0.431, *p* = 0.058 and Pearson’s r: 0.396, *p* = 0.075, respectively).

For all patients, the mean new/enlarged T2 lesions were 0.23 ± 0.16 and 0.23 ± 0.16 at 12 and at 24 months, respectively. For responders at 24 months, the mean new/enlarged T2 lesions were 0.15 ± 0.15 at 12 and 0 at 24 months. For non-responders at 24 months, the mean new/enlarged T2 lesions were 0.33 ± 0.33 and 0.56 ± 0.38 at 12 and at 24 months, respectively. The mean number of new/enlarged T2 lesions did not differ at 12 and at 24 months between the two groups (*p* = 0.896 and *p* = 0.393, respectively). Moreover, the mean change in new/enlarged T2 lesions from baseline did not differ between the two groups at the 12-month point estimate (mean change in new/enlarged T2 lesions −1 ± 0.42 for responders at 24 months vs. −0.56 ± 0.58 for non-responders at 24 months, *p* = 0.601), as well as at the 24-month point estimate (mean change in new/enlarged T2 lesions −1.15 ± 0.36 for responders at 24 months vs. −0.33 ± 0.37 for non-responders at 24 months, *p* = 0.144). The mean change in new/enlarged T2 lesions between 12 and 24 months was −0.15 ± 0.15 for responders at 24 months vs. 0.22 ± 0.55 for non-responders at 24 months, *p* = 0.556) (Figure 2B). Time did not have a significant effect on mean new/enlarged T2 lesions, evaluated with a general linear model in a repeated-measures setting (F = 0.324, *p* = 0.725). In this model, the baseline MRI activity, defined as new/enlarged T2 and Gd(+) lesions on the brain and cervical MRI, exhibited a significant effect on the mean change of new/enlarged T2 lesions over time (F = 17.224, *p* < 0.001). Similarly, time did not have a significant effect on the mean new/enlarged T2 lesions when group allocation based on response at 24 months was taken into account in the model as a between-subjects factor (F = 0.501, *p* = 0.61). In this model, the baseline MRI activity, defined as new/enlarged T2 and Gd(+) lesions on the brain and cervical MRI, exhibited a significant effect on the mean change of new/enlarged T2 lesions over time (F = 16.877, *p* < 0.001).

- Cognitive function

The mean scores in cognitive testing and self-reported fatigue for baseline, as well as for 12 and 24 months, are exhibited in Table 3.

The mean values for SDMT, GVLT, BVMT-R and MFIS did not differ at baseline between responders at 24 months vs. non-responders at 24 months. A tendency toward increased mean SDMT values at baseline, at 12 and at 24 months, was evident for responders vs. non-responders (47.11 ± 3.21 vs. 38.62 ± 3.14, *p* = 0.096 at baseline; 45 ± 3.37 vs. 36.38 ± 2.6, *p* = 0.06 at 12 months and 46.67 ± 3.18 vs. 38.08 ± 2.79, *p* = 0.071 at 24 months). At point estimates, the mean change from baseline was comparable for SDMT, GVLT, BVMT-R and MFIS at 12 and at 24 months. Time did not have a significant effect on the mean SDMT scores evaluated with a general linear model in a repeated-measures setting, neither when all patients were considered together (F = 0.426, *p* = 0.538) nor when group allocation based on response at 24 months was taken into account in the model as a between-subjects factor (F = 0.401, *p* = 0.598). Similar trends were identified for the mean GVLT scores (F = 0.556, *p* = 0.525 and F = 0.539, *p* = 0.534, respectively), mean BVMT-R scores (F = 0.525, *p* = 0.596 and F = 0.486, *p* = 0.62, respectively) and mean MFIS scores (F = 2.119, *p* = 0.137 and F = 2.056, *p* = 0.146, respectively).

- MRI volumetry—association with cognitive performance

**Figure 3:**
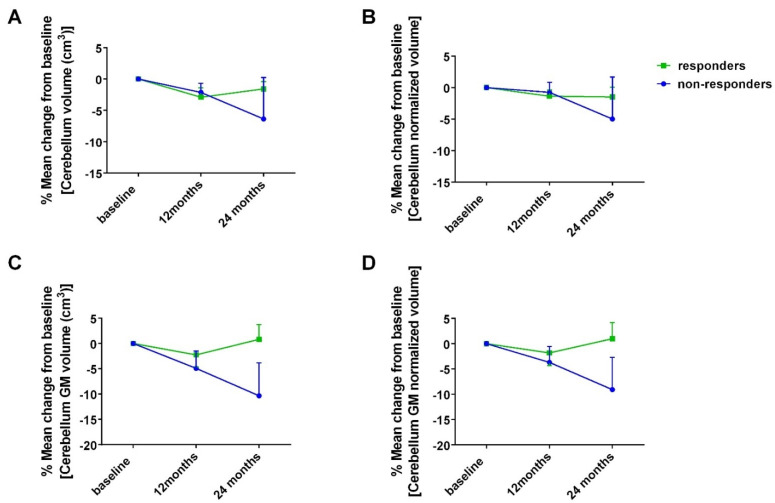
Mean % change in cerebellar volumetry parameters for responders at 24 months vs. non-responders at 24 months. (**A**) absolute cerebellar volume (cm3); (**B**) normalized cerebellar volume; (**C**) absolute gray matter cerebellar volume (cm^3^); (**D**) normalized gray matter cerebellar volume. Dots and error bars represent mean values and standard error of mean, respectively. GM: Gray Matter.

MRI volumetric and lesion load analysis at baseline, at 12 and at 24 months, for all patients, as well as for responders at 24 months vs. non-responders at 24 months, are presented in Supplementary Table S1. For two patients, one responder and one non-responder at 24 months, MRI analysis was not performed due to moving artifacts. At point estimates, the mean MRI volumetry and lesion analysis parameters did not differ between responders at 24 months vs. non-responders at 24 months, with the exception of cerebellar volumetric measurements (Supplementary Table S1). Although the mean % change in cerebellar volume and gray matter cerebellar volume at point estimates did not differ between responders and non-responders at 24 months, (Supplementary Table S2 and Figure 3), time, overall, exhibited a tendency toward a significant effect on the mean cerebellar volume, both for absolute and normalized values, evaluated with a general linear model in a repeated-measures setting for all patients (F = 3.684, *p* = 0.065 and F = 3.372, *p* = 0.083 for absolute and for normalized values, respectively), as well as when group allocation based on response at 24 months was taken into account in the model as a between-subjects factor (F = 3.729, *p* = 0.065 and F = 3.285, *p* = 0.088 for absolute and for normalized values, respectively). This effect was more prominent for mean gray matter cerebellar volume for all patients (F = 4.342, *p* = 0.23 and F = 4.279, *p* = 0.024 for absolute and for normalized values, respectively), as well as when group allocation based on response at 24 months was taken into account in the model as a between-subjects factor (F = 5.082, *p* = 0.014 and F = 4.862, *p* = 0.016 for absolute and for normalized values, respectively) (Supplementary Table S1).

In the overall cohort, hierarchical linear models (HLM) with repeated-measures data in a mixed setting elucidated several volumetry and lesion analysis parameters that exhibited an association of variability with cognitive and EDSS scores’ variability over time (Supplementary Table S3). Overall, lateral ventricles’ volume variability exhibited an association with the variability of SDMT, GVLT, BVLT-R and MFIS scores over time. Moreover, lesion volumetry parameters differentially exhibited association with SDMT, GVLT, BVLT-R and/or MFIS scores. However, a single lesion volumetry parameter universally exhibiting an association with cognitive scores was not identified. Cerebellum and cerebellum gray matter volumetric parameters exhibited an association with SDMT scores. Thalamus volumetric parameters exhibited an association of variability with GVLT and BVLT-R scores over time.

When taking into account clinically meaningful change in SDMT as a ≥4-point increase (improvement) or a ≥4-point reduction (deterioration), there was no difference in the frequency of patients who exhibited either improved or deteriorated SDMT scores between responders vs. non-responders at 12 months (SDMT improved vs. non-improved 2/11 for responders vs. 1/8 for non-responders, *p* = 0.774 at 12 months; SDMT improved vs. non-improved 4/9 for responders vs. 2/7 for non-responders, *p* = 0.658 at 24 months; SDMT deteriorated vs. non-deteriorated 6/7 for responders vs. 2/7 for non-responders, *p* = 0.251 at 12 months; SDMT deteriorated vs. non-deteriorated 5/8 for responders vs. 2/7 for non-responders, *p* = 0.421 at 24 months). Cerebellar volume loss at 12 months was greater for patients with clinically meaningful deterioration in SDMT at 24 months, both with respect to absolute volume loss (SDMT-deteriorated vs. SDMT non-deteriorated −8.55 ± 2.87 vs. −1.2 ± 0.99, *p* = 0.05) and (%) volume loss (SDMT-deteriorated vs. SDMT non-deteriorated −6.52 ± 2.39 vs. −0.93 ± 0.8, *p* = 0.01). Similarly, for the gray matter cerebellar volume at 12 months, there was a tendency toward greater loss for patients with clinically meaningful deterioration in SDMT at 24 months, both with respect to absolute volume loss (SDMT-deteriorated vs. SDMT non-deteriorated −10.29 ± 6.07 vs. −0.92 ± 1.73, *p* = 0.06) and normalized volume loss (SDMT-deteriorated vs. SDMT non-deteriorated −9.06 ± 4.29 vs. −0.88 ± 1.83, *p* = 0.052). Moreover, when clinically meaningful SDMT deterioration at 12 months was taken into account, cerebellar gray matter volume loss at 12 months was greater for patients with clinically meaningful deterioration in SDMT at 12 months, both with respect to absolute volume loss (SDMT-deteriorated vs. SDMT non-deteriorated −10.79 ± 5.88 vs. −0.7 ± 1.74, *p* = 0.041) and normalized volume loss (SDMT-deteriorated vs. SDMT non-deteriorated −9.14 ± 3.88 vs. −0.84 ± 1.97, *p* = 0.048). No difference with respect to change in volumetric parameters was evident between patients with and without clinically meaningful SDMT improvement at 12 and at 24 months (data not shown).

- Immune cell phenotype

**Figure 4:**
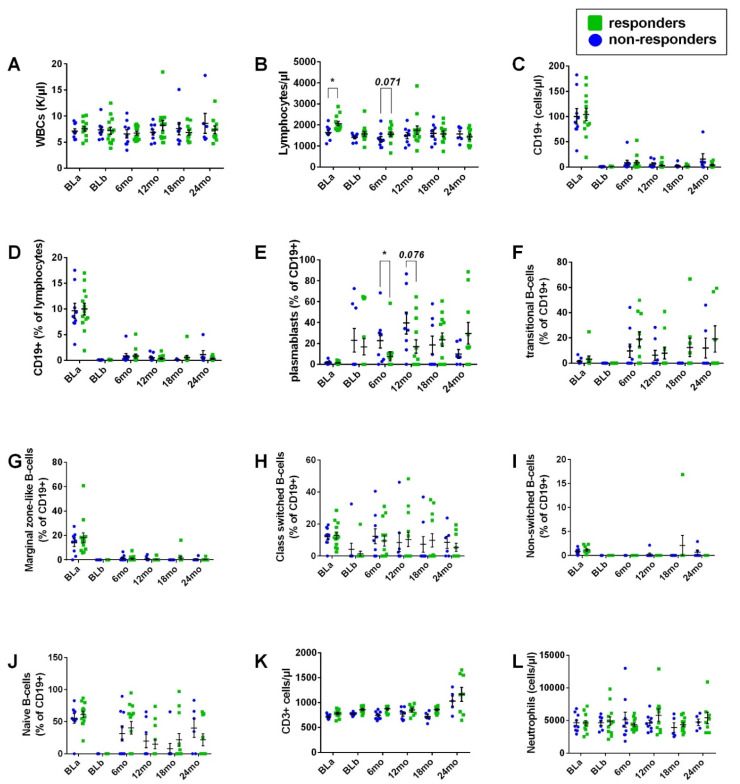
Immune cell phenotype for responders at 24 months vs. non-responders at 24 months. White blood cells (**A**), lymphocytes (**B**), CD19+ cells (absolute numbers and relative % frequency of lymphocytes, (**C**,**D**), respectively), plasmablasts (**E**), transitional B-cells (**F**), marginal-zone-like B-cells (**G**), class-switched memory B-cells (**H**), non-switched memory B-cells (**I**), naïve B-cells (**J**), CD3+ cells (**K**) and neutrophils (**L**) assessed at baseline pre-first infusion (BLa) and 15 days after treatment with the first infusion and pre-second infusion (BLb), as well as in 6-month intervals thereafter, prior to the next scheduled infusion of ocrelizumab. Dots and error bars represent mean values and standard error of mean, respectively. * *p* < 0.05.

B-cell immunophenotype is exhibited in Figure 4 and in Supplementary Table S4. Overall, ocrelizumab infusion resulted in a striking peripheral blood (PB) reduction in the mean absolute numbers and relative % frequency of CD19+ cells, as well as in the relative % frequency of transitional B-cells, marginal-zone-like B-cells, class-switched memory B-cells and naïve B-cells. Total white blood cells, CD3+ cells and neutrophils remained largely unaffected. An increase in the relative % frequency of plasmablasts was evident over time. Transitional B-cells, class-switched B-cells and naïve B-cells re-emerged in subsequent follow-up evaluations, as was also evident for CD19+ cells. At point estimates, white blood cells, lymphocytes, CD19+ cells (absolute numbers and relative % frequency of lymphocytes), transitional B-cells, marginal-zone-like B-cells, class-switched memory B-cells, non-switched memory B-cells, naïve B-cells, as well as CD3+ cells and neutrophils did not differ between responders at 24 months vs. non-responders at 24 months. However, responders at 24 months exhibited significantly reduced plasmablasts ((%) of CD19+ cells) compared to non-responders at 24 months, at the 6-month point estimate (7.69 ± 4.4 vs. 22.66 ± 7.19, respectively, *p* = 0.043) and a similar tendency at the 12-month point estimate (17.05 ± 6.46 vs. 39.54 ± 10.62, respectively, *p* = 0.076). The emergence of plasmablasts in the non-responder population appeared to be independent of the prior-to-treatment lymphocyte cell counts and the effectiveness of lymphocyte depletion, as evidenced by increased PB absolute lymphocyte cell counts (cells/μL) in the responders before treatment initiation compared to the non-responders (2065.38 ± 99.12 vs. 1647.78 ± 110.25, respectively, *p* = 0.011) and by a tendency toward increased absolute lymphocyte cell counts (cells/μL) in responders at the 6-month point estimate compared to non-responders (1565.38 ± 104.65 vs. 1316.67 ± 129.91, respectively, *p* = 0.071).

- Serum cytokine analysis

**Figure 5:**
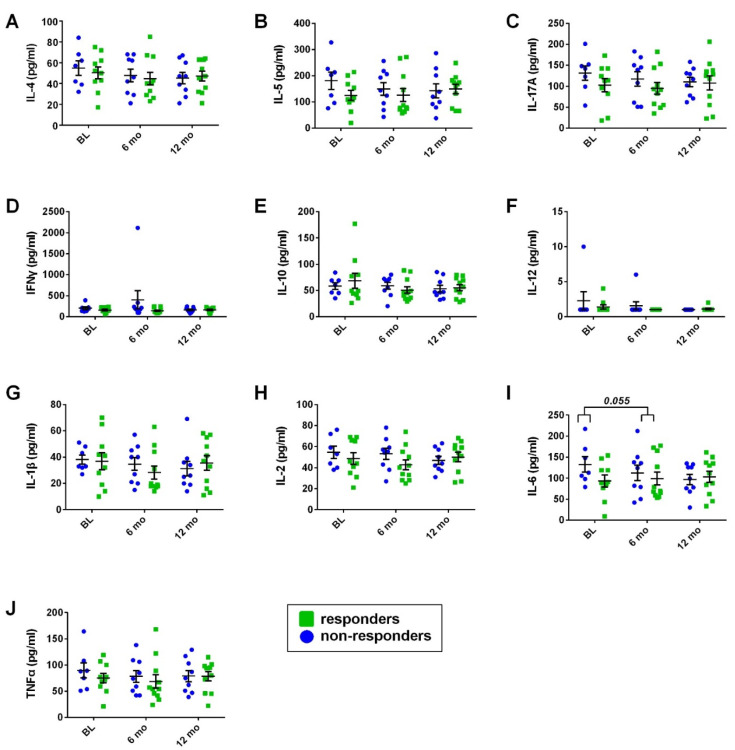
Serum cytokine profile for responders at 24 months vs. non-responders at 24 months. Interleukin-4 (**A**), Interleukin-5 (**B**), Interleukin-17A (**C**), Interferon-γ (**D**), Interleukin-10 (**E**), Interleukin-12 (**F**), Interleukin-1β (**G**), Interleukin-2 (**H**), Interleukin-6 (**I**), Tumor Necrosis Factor-α (**J**) assessed at baseline pre-first infusion (BL), as well as in 6-month intervals thereafter, prior to the next scheduled infusion of ocrelizumab. The brackets indicate the difference in % mean change from baseline for Interleukin-6 serum levels between responders at 24 months vs. non-responders at 24 months. IL: Interleukin; IFNγ: Interferon-γ; TNFα: Tumor Necrosis Factor-α. Dots and error bars represent mean values and standard error of mean, respectively.

Overall, responders at 24 months did not exhibit differences in their serum cytokine levels and/or % mean change from baseline in their cytokine levels at point estimates when compared to non-responders at 24 months (Figure 5 and Supplementary Table S5). An exception to this finding was IL-6 levels, for which a tendency toward a difference in the % mean change from baseline was evident at the 12-month point estimate between responders at 24 months vs. non-responders at 24 months. Similarly, time exhibited a tendency toward a significant effect on mean IL-6 scores evaluated with a general linear model in a repeated-measures setting when group allocation based on response at 24 months was taken into account in the model as a between-subjects factor (F = 2.868, *p* = 0.072). More specifically, non-responders at 24 months exhibited IL-6 reduction at 6- and 12-month point estimates compared to baseline, whereas for responders at 24 months, IL-6 levels were comparable across the time points (Figure 5 and Supplementary Table S5).

## 4. Discussion

Ocrelizumab has been reported to exhibit beneficial effect not only in patients with RRMS but also in patients with active PPMS [29,30], thus becoming the first DMT available for the latter form of the disease. Anti-CD20 treatment evidently eliminates B-cells acting as antigen-presenting cells [63]. Moreover, as B-cells produce pro- and anti-inflammatory cytokines, anti-CD20 treatment is expected to lead into alterations in the serum cytokine profile, thus interfering with T-cell activation status and ameliorating disease-mediating T-cell responses [63,64]. Notably, ocrelizumab-induced clinical benefit seems to be more prominent for patients with CNS inflammatory lesions, as indicated by the presence of MRI activity. In this respect, the individual response to ocrelizumab remains to be fully characterized, thus underlining the need for the identification of clinically relevant immunological markers. In the present study, we aimed to assess the efficacy of ocrelizumab in a cohort of patients with active progressive MS and to explore differences with respect to clinical, radiological and laboratory parameters in patients with optimal vs. sub-optimal treatment response.

In our study, baseline EDSS and self-reported fatigue, as measured with MFIS, were significantly correlated with volumetry parameters of the brainstem, thalamus and the cerebellum, whereas they exhibited a tendency to correlate with overall lesion burden parameters. Time, overall, exhibited a tendency toward a significant effect on mean cerebellar volume for all patients, as well as in relation to the group allocation based on response at 24 months. This effect was more prominent for mean gray matter cerebellar volume for all patients, as well as when group allocation based on response at 24 months was taken into account. More specifically, responders at 24 months maintained lower cerebellar volume loss and lower gray matter cerebellar volume loss over time compared to non-responders at 24 months. This result is in accordance with recently announced data on the effect of ocrelizumab treatment on cerebellar volume loss based on a sub-analysis of the ORATORIO study [65]. With regard to cognitive performance, no clinically significant difference was observed in the performance between the two patient groups. However, in all patients, a decrease in the performance in processing speed, as measured using SDMT, was correlated with greater cerebellar volume loss. The relation of SDMT performance with cerebellar pathology in MS has been demonstrated in multiple studies [66,67,68,69]. Cerebellum is interconnected with many cortical areas involved in cognition, such as the prefrontal cortex and the lateral parietal cortex [70,71]. It seems that damage in the cerebellum may lead to dysfunction of circuits involved in attention, processing speed and executive functions, among others [72,73].

The proportion of MRI efficacy, defined as absence of new/enlarged T2 lesions on the brain and cervical MRI, was >90% in 12- and 24-month point estimates for the overall cohort, thus referring to both responders and non-responders. Of note, the number of new/enlarged T2 lesions was indicated upon routine MRI evaluation, relative to the last previous MRI, and independently confirmed by the treating neurologists of the study group. However, as indicated by the lesion volume measurements derived from volumetry analysis, patients defined as non-responders exhibited an overall increase in the mean lesion volume measurements, whereas patients defined as responders exhibited an overall decrease in the mean lesion volume measurements. Although the repeated-measures general linear model mean comparison between responders and non-responders for these measurements did not reach statistical significance, we believe that this discrepancy between the routine MRI evaluation and the lesion volumetry measurements underlines the need for more thorough MRI evaluation, even with the integration of volumetric and lesion-volumetric analyses in the disease management algorithms, an especially prominent need in progressive MS (reviewed in Ref [74]). Validation and refinement of the MRI volumetry protocols in order to better adhere to clinical decision making is necessary and requires large-cohort prospective studies.

As expected, ocrelizumab infusion resulted in a striking reduction in most PB B-cell subtypes, whereas white blood cells, CD3+ cells and neutrophils remained largely unaffected. Additionally, consistent with existing studies, an increase in the relative % frequency of plasmablasts was evident over time [32,33]. Most B-cell subtypes at point estimates did not differ between responders at 24 months vs. non-responders at 24 months in absolute and proportional values. However, responders at 24 months exhibited significantly reduced PB plasmablasts ((%) of CD19+ cells) compared to non-responders at 24 months at the 6-month point estimate and a similar tendency at the 12-month point estimate. Increased plasmablasts both at baseline and during treatment may indicate sub-optimal response to anti-CD20 treatment in rheumatoid arthritis (RA) [57,75] and in systemic lupus erythematosus (SLE) [76]. To our knowledge, reduced plasmablast depletion efficacy has been linked for the first time to sub-optimal response to anti-CD20 treatment in active progressive MS. This finding needs to be further validated in a large-cohort study. Of note, insufficient memory B-cell depletion has been linked to poor treatment outcomes in patients with neuromyelitis optica (NMO) under rituximab [77].

In systemic rheumatoid disease, increased serum IL-6 levels prior to treatment onset have been linked to poor treatment response, and the persistence of IL-6 levels during treatment has been advocated as a biomarker of resistance to treatment [78]. Of note, the effect of IL-6 on MS is poorly characterized, and existing evidence indicates that IL-6 may be both harmful and beneficiary for MS. IL-6, together with TGFβ, is required for Th17- T-effector phenotype induction, and IL-6 may attenuate the production of T-regulatory cells [79]. Although IL-6 appears to promote EAE pathology in an experimental setting, this finding is poorly translated in MS, as IL-6 may also exhibit immunosuppressive properties [80,81]. In fact, existing evidence indicates that administration of IL-6 blocking pharmaceutical agents in the context of rheumatoid disease may precipitate CNS demyelination [82,83]. In the present study, ocrelizumab response was linked neither to low IL-6 serum levels at baseline nor to IL-6 level reduction during treatment compared to baseline values, thus underlying the assumption that the effect of anti-CD20 cell depletion in MS may be independent of IL-6 regulation.

### Limitations and Future Directions

Our study is subjected to limitations. First, low participant numbers may have accounted for insufficient statistical power to elucidate differences in the clinical outcomes and the laboratory findings between responders vs. non-responders. This is an inherent limitation of a real-world one-center study. Of note, the results elucidated in the present study need to be validated in a larger cohort.

Second, T-cell immunophenotype analysis was not conducted in the frame of the present study. T-cells are the main immune cellular elements that confer damage in the target organ, namely, the CNS, in MS. It has therefore been advocated that B-cell depletion treatment may induce beneficial effect in MS clinical outcomes via eliminating the B-cell compartment that essentially primes antigen presentation and the subsequent T-cell clonal expansion and T-effector lineage commitment in response to antigen-specific stimuli. In this respect, a study of T-cell immunophenotype alterations may accurately dissect the mechanisms by which B-cell depletion is beneficiary in a subset of patients with MS, otherwise traditionally regarded as a T-cell-mediated disease. Moreover, anti-CD20 pharmacological agents have been shown to eliminate not only CD20-bearing B-cells but also specific CD3+CD20^dim^, CD8+CD20+ and other T-cell subsets [32,33,34,84] with an evidently pathogenic potential in the context of MS.

Third, regarding the volumetry analysis followed and the possible associations with cognitive measurements, it should be noted that the VolBrain algorithm provides a report value for cerebrum gray matter volume without explicitly defining cortical gray matter volume. A reduction in the rate of global and regional brain atrophy has been linked to response to ocrelizumab [74]. In this respect, a more thorough investigation and volumetry analysis protocol by a specialized neuro-radiologist, according to the SIENA protocol, would be beneficial in the frame of the present study in order to fulfill the requirement for a cortical gray matter analysis. In line with this fact, the possibility of an association between cortical gray matter changes and the changes in the cognition cannot be fully assessed. We consider this an inherent limitation of our study on the basis of the volumetry analysis tool that was used.

Of note, we considered as responders to treatment those patients who did not present progression of disability under ocrelizumab, thus signifying optimal response in the context of active progressive MS. However, for active progressive MS, a well-defined, universally applied consensus for treatment response remains to be described. In this respect, one may also consider the reduction rate of cumulative disability as an approach to assess treatment response, taking into account the fact that plateaus in the disease progression may occur during the natural course of the disease [62]. The clinically meaningful threshold for the reduction in the disability progression rate, regarded as treatment response in the context of active progressive MS, should be explored in future studies that also take into consideration the rate of disability progression before treatment initiation. Other structural biomarkers, such as the rate of retinal inner nuclear layer thinning, assessed with optical coherence tomography (OCT) techniques, are also under evaluation and may collectively contribute to an effective assessment of the neurodegenerative component in progressive MS [85].

Moreover, recent evidence regarding the effects of ocrelizumab on fluid biomarkers suggests that serum neurofilament light chain levels are also reduced as a result of the treatment, an observation with implication for a possible treatment effect on axonal damage and loss in the frame of progressive MS [74]. Additional serum biomarkers currently under study may contribute to the development and validation of a consensus guidance regarding the assessment of treatment efficacy and the optimal treatment administration frequency in patients with aPPMS under ocrelizumab.

## 5. Conclusions

In the present study, the efficacy of ocrelizumab in a cohort of patients with active progressive MS was assessed, and clinical, radiological and laboratory attributes were linked to treatment response. With respect to radiological biomarkers, the response to ocrelizumab was linked to lower cerebellar volume loss and lower gray matter cerebellar volume loss over time. Moreover, to our knowledge, reduced plasmablast depletion efficacy was linked for the first time to sub-optimal response to anti-CD20 treatment in active progressive MS, and this effect may be independent of IL-6 regulation. Further studies are necessary in order to enhance existing knowledge regarding the biological implications and the clinical outcomes that are associated with the effect of ocrelizumab on the immunological profile in the context of active PPMS.

## Figures and Tables

**Table 1:** Patients’ baseline characteristics (all patients and non-responders at 24 months vs. responders at 24 months).

	All (N = 22)	Non-Responders (N = 9)	Responders (N = 13)	*p* *
Gender (male/female) Age Education (years)	12/10	5/4	7/6	0.937
	48.5 ± 1.69	49.56 ± 3.45	47.77 ± 1.69	0.948
	14.05 ± 0.7	15.44 ± 1.16	13 ± 0.78	0.058
Disease duration (years) Time from diagnosis (years)	9.05 ± 0.92	8.89 ± 1.79	9.15 ± 1.02	0.556
	5.59 ± 0.84	6.44 ± 1.44	6.69 ± 1.06	0.512
Smoking (yes/no) Cardiovascular comorbidities ** Other (non-cardiovascular) comorbidities *** EDSS	10/12	2/7	8/5	0.069
	16/2/4	8/0/1	8/2/3	0.309
	16/6/1/1/1	7/2/0/0/0	9/4/1/1/1	0.556
	4.91 ± 0.3	4.56 ± 0.56	5.15 ± 0.34	0.324
MRI new/enlarged T2 lesions (brain and cervical) MRI Gd+ lesions (brain and cervical)	1.05 ± 0.26	0.89 ± 0.39	1.15 ± 0.36	0.647
	0.36 ± 0.12	0.44 ± 0.18	0.31 ± 0.17	0.512

Yrs., years; EDSS, Expanded Disability Status Scale; MRI, Magnetic Resonance Imaging; * for the comparison of non-responders at 24 months vs. responders at 24 months; ** none/dyslipidemia/hypertension; *** none/affective disorder/hypothyroidism/migraine/chronic hepatitis B (>1 other comorbidity for three patients); values represent mean ± standard error of mean or number of patients; *p* values for Mann–Whitney U-Test or Pearson’s chi-square test, where applicable.

**Table 2:** Proportion of responders and patients free from events defined as secondary outcomes, at 12 and 24 months.

Proportion of Patients, n/N (%) 95% CI	12 Months (N = 22)	24 Months (N = 22)
Responders	14/22 (63.6%) 0.41–0.83	13/22 (59.1%) 0.36–0.79
No CDA	14/22 (63.6%) 0.41–0.83	13/22 (59.1%) 0.36–0.79
No new/enlarged T2 lesions (brain and cervical MRI)	20/22 (90.9%) 0.71–0.99	20/22 (90.9%) 0.71–0.99

CI, confidence intervals (Clopper–Pearson exact method); CDA, Confirmed Disability Accumulation; MRI, Magnetic Resonance Imaging.

**Table 3:** Patients’ cognitive assessment (all patients and non-responders at 24 months vs. responders at 24 months).

	All (N = 22)	Non-Responders (N = 9)	Responders (N = 13)	*p* *
SDMT				
baseline	42.09 ± 2.4	47.11 ± 3.21	38.62 ± 3.14	0.096
12 months	39.91 ± 2.21	45 ± 3.37	36.38 ± 2.6	0.06
24 months	41.59 ± 2.25	46.67 ± 3.18	38.08 ± 2.79	0.071
change from baseline (12 months)	−2.18 ± 0.89	−2.11 ± 1.45	−2.23 ± 1.17	0.061
change from baseline (24 months)	−5 ± 1.28	−0.44 ± 1.45	−0.54 ± 1.96	0.253
GVLT				
baseline	52.82 ± 2.33	53.56 ± 3.2	52.31 ± 3.37	0.556
12 months	54.91 ± 2.52	58.11 ± 3.73	52.69 ± 3.37	0.324
24 months	55.73 ± 2.43	60.33 ± 3.34	52.54 ± 3.2	0.096
change from baseline (12 months)	2.09 ± 2.09	4.56 ± 3.04	0.38 ± 2.83	0.058
change from baseline (24 months)	2.91 ± 2.43	6.78 ± 2.81	0.23 ± 3.53	0.288
BVMT-R				
baseline	19.14 ± 1.7	20.89 ± 1.94	17.92 ± 2.56	0.647
12 months	20.41 ± 1.95	22.56 ± 2.87	18.92 ± 2.64	0.357
24 months	18.45 ± 1.78	21.89 ± 2.55	16.08 ± 2.29	0.126
change from baseline (12 months)	1.27 ± 1.57	1.67 ± 2.67	1 ± 2	0.338
change from baseline (24 months)	−0.68 ± 1.64	1 ± 2.44	−1.85 ± 2.22	0.165
MFIS				
baseline	36.32 ± 3.2	30.22 ± 3.94	40.54 ± 4.42	0.082
12 months	37.95 ± 3.86	33.89 ± 7.15	40.77 ± 4.34	0.512
24 months	41.55 ± 3.64	36.11 ± 6.09	45.31 ± 4.4	0.235
change from baseline (12 months)	1.64 ± 3.56	3.67 ± 7.17	0.23 ± 3.66	0.173
change from baseline (24 months)	5.23 ± 3.07	5.89 ± 5.96	4.77 ± 3.38	0.12

SDMT, Symbol Digit Modalities Test; GVLT, Greek Verbal Learning Test; BVMT-R, Brief Visuospatial Memory Test-Revised; MFIS, Modified Fatigue Impact Scale; * for the comparison of non-responders at 24 months vs. responders at 24 months (for the comparison at point estimates: Mann–Whitney U-test; for the comparison of mean change from baseline: repeated-measures general linear model); values represent mean ± standard error of mean.

## Data Availability

Anonymized data not published within this article will be made available on request from any qualified investigator. The principal author has full access to the data used in the analyses in the manuscript. The principal author takes full responsibility for the data, the analyses and interpretation, and the conduct of the research; they have full access to all of the data; they have the right to publish any and all data, separate and apart from the guidance of any sponsor.

## Supplementary Materials

The following supporting information can be downloaded at: www.mdpi.com/article/10.3390/cells11121959/s1, Table S1: MRI volumetry and lesion analysis parameters at baseline, at 12 and at 24 months for all patients and for responders at 24 months vs. non-responders at 24 months; Table S2: % mean reduction in cerebellar volume and in gray matter cerebellar volume from baseline at 12 and at 24 months for responders at 24 months vs. non-responders at 24 months; Table S3: Association of volumetry and lesion analysis parameters’ variability with cognitive and EDSS scores’ variability over time (only associations with *p* values < 0.1 are presented); Table S4: Immune cell phenotype assessed at baseline pre-first infusion (BLa) and 15 days after treatment with the first infusion and pre-second infusion (BLb), as well as in 6-month intervals thereafter, prior to the next scheduled infusion of ocrelizumab and % change from baseline for 6-month interval point estimates for all patients and non-responders at 24 months vs. responders at 24 months; Table S5: Cytokine levels assessed at baseline pre-first infusion (BL), as well as in 6- and 12-month time points, prior to the next scheduled infusion of ocrelizumab and % mean cytokine change from baseline for 6- and 12-month time points for all patients and non-responders at 24 months vs. responders at 24 months.
