# Supplementary material for: Ocrelizumab in Patients with Active Primary Progressive Multiple Sclerosis: Clinical Outcomes and Immune Markers of Treatment Response
Source: Cells. 2022 Jun 17;11(12):1959. doi: 10.3390/cells11121959 (PMC9222195; doi:10.3390/cells11121959)
Supplement: Supplementary file 1 [file cells-11-01959-s001.zip › cells-1761513-supplementary.pdf]

Table S1. MRI Volumetry and Lesion analysis parameters at baseline, at 12 and at 24 months for all patients and for responders at 24 months vs. non-responders at 24 months

Parameter	All patients (N=20)		Non-responders (N=8)		Responders (N=12)			Effect of time (all patients)		Effect of time (Responders vs. Non-responders)	
	Mean	SE	Mean	SE	Mean	SE	p*	F	p	F	p
WM cm3 BL	456,83	19,18	487,18	31,93	436,59	23,10	0,208	1,82	0,181	1,809	0,198
WM cm3 12mo	437,80	15,90	463,32	27,62	420,80	18,40	0,157				
WM cm3 24mo	467,82	30,23	523,54	67,39	430,67	19,74	0,181				
WM % BL	32,56	1,16	34,09	1,85	31,55	1,48	0,208	1,811	0,182	1,775	0,189
WM % 12mo	31,29	0,83	32,68	1,53	30,36	0,90	0,27				
WM % 24mo	33,37	1,82	36,51	4,14	31,27	1,11	0,343				
GM cm3 BL	711,31	31,01	701,69	50,65	717,72	40,88	0,571	1,529	0,234	1,419	0,26
GM cm3 12mo	734,00	22,10	719,28	44,59	743,82	23,24	0,343				
GM cm3 24mo	708,96	21,27	683,29	38,31	726,08	24,76	0,571				
GM % BL	50,76	1,93	49,29	3,48	51,74	2,32	0,343	0,935	0,404	0,864	0,433
GM % 12mo	52,80	1,62	50,97	3,11	54,02	1,77	0,27				
GM % 24mo	51,00	1,52	48,27	3,07	52,83	1,38	0,305				
CSF cm3 BL	233,29	21,22	236,54	35,72	231,13	27,46	0,91	3,086	0,062	2,888	0,074
CSF cm3 12mo	224,00	24,52	230,72	39,43	219,52	32,67	0,91				
CSF cm3 24mo	219,34	22,79	216,24	39,07	221,40	29,10	0,851				
CSF % BL	16,67	1,50	16,62	2,47	16,71	1,98	0,678	2,616	0,091	2,46	0,105
CSF % 12mo	15,91	1,65	16,35	2,75	15,62	2,15	0,851				
CSF % 24mo	15,63	1,55	15,22	2,73	15,91	1,93	0,734				
Brain cm3 BL	1170,09	32,55	1188,87	44,11	1157,57	46,79	0,792	2,986	0,067	2,829	0,077
Brain cm3 12mo	1171,81	28,10	1182,60	47,46	1164,61	36,13	0,734				
Brain cm3 24mo	1176,79	29,81	1206,83	47,26	1156,76	38,99	0,624				
Brain % BL	83,33	1,50	83,38	2,47	83,29	1,98	0,678	2,616	0,091	2,46	0,105
Brain % 12mo	84,09	1,65	83,65	2,75	84,38	2,15	0,851				
Brain % 24mo	84,37	1,55	84,78	2,73	84,09	1,93	0,734				
IC cm3 BL	1401,42	28,33	1425,41	28,79	1385,44	43,59	0,521	1,095	0,348	1,044	0,366

IC cm3 12mo	1395,81	25,55	1413,32	29,36	1384,13	38,51	0,734				
IC cm3 24mo	1396,12	27,01	1423,07	26,72	1378,16	41,52	0,521				
Cerebrum cm3 BL	1012,77	28,43	1028,59	36,02	1002,22	41,85	0,734				
Cerebrum cm3 12mo	1018,09	24,66	1025,88	40,02	1012,90	32,63	0,734	3,01	0,065	2,885	0,074
Cerebrum cm3 24mo	1026,03	28,70	1057,67	49,62	1004,94	35,01	0,624				
Cerebrum % BL	72,25	1,34	72,15	2,02	72,32	1,85	0,851				
Cerebrum % 12mo	73,07	1,49	72,58	2,34	73,40	2,01	0,792	2,737	0,082	2,602	0,093
Cerebrum % 24mo	73,55	1,55	74,27	2,90	73,06	1,80	0,91				
Cerebrum GM cm3 BL	605,58	27,04	594,30	42,55	613,10	36,38	0,521				
Cerebrum GM cm3 12mo	625,57	19,59	612,84	38,12	634,05	21,73	0,343	1,933	0,163	1,792	0,187
Cerebrum GM cm3 24mo	608,08	17,44	589,93	27,98	620,18	22,59	0,624				
Cerebrum GM % BL	43,21	1,69	41,75	2,93	44,18	2,08	0,27				
Cerebrum GM % 12mo	45,00	1,43	43,43	2,66	46,04	1,63	0,27	1,302	0,288	1,201	0,317
Cerebrum GM % 24mo	43,72	1,22	41,65	2,32	45,11	1,26	0,384				
Cerebrum WM cm3 BL	407,19	16,35	434,29	26,96	389,12	19,68	0,27				
Cerebrum WM cm3 12mo	392,53	13,31	413,04	23,32	378,85	15,39	0,181	1,772	0,188	1,782	0,203
Cerebrum WM cm3 24mo	417,95	27,25	467,74	61,86	384,76	16,23	0,27				
Cerebrum WM % BL	29,04	0,99	30,41	1,57	28,13	1,26	0,208				
Cerebrum WM % 12mo	28,07	0,69	29,14	1,28	27,36	0,76	0,427	1,763	0,19	1,749	0,194
Cerebrum WM % 24mo	29,82	1,65	32,63	3,82	27,96	0,92	0,384				
Cerebelum cm3 BL	132,86	4,07	136,82	7,67	130,23	4,60	0,624				
Cerebelum cm3 12mo	131,76	3,68	134,83	7,48	129,72	3,78	1	0,834	0,445	0,93	0,407
Cerebelum cm3 24mo	128,96	3,84	127,53	8,00	129,91	3,88	0,97				
Cerebelum % BL	9,50	0,20	9,60	0,46	9,44	0,16	1				
Cerebelum % 12mo	9,44	0,21	9,53	0,45	9,39	0,19	0,792	0,381	0,687	0,46	0,637
Cerebelum % 24mo	9,26	0,24	8,98	0,56	9,44	0,17	1				
Cerebelum GM cm3 BL	96,87	3,91	98,94	7,76	95,49	4,25	0,792				
Cerebelum GM cm3 12mo	99,14	3,13	97,75	6,89	100,06	2,80	0,157	1,308	0,286	1,299	0,29
Cerebelum GM cm3 24mo	91,83	4,81	85,79	11,22	95,86	3,06	0,427				
Cerebelum GM % BL	6,93	0,25	6,96	0,53	6,91	0,25	0,678				
Cerebelum GM % 12mo	7,13	0,23	6,93	0,49	7,27	0,22	0,238	1,01	0,377	1,025	0,373
Cerebelum GM % 24mo	6,63	0,35	6,08	0,80	6,99	0,21	0,181				
Cerebelum WM cm3 BL	35,99	2,96	37,88	5,87	34,73	3,20	0,97	1,238	0,305	1,144	0,334

Cerebelum WM cm3 12mo	32,63	2,61	37,08	5,06	29,66	2,61	0,208				
Cerebelum WM cm3 24mo	37,13	3,28	41,73	6,34	34,06	3,43	0,427				
Cerebelum WM % BL	2,55	0,19	2,64	0,37	2,50	0,22	1				
Cerebelum WM % 12mo	2,31	0,16	2,60	0,32	2,12	0,15	0,238	1,14	0,334	1,052	0,363
Cerebelum WM % 24mo	2,63	0,21	2,90	0,40	2,45	0,22	0,571				
Brainstem cm3 BL	22,12	0,86	22,57	1,42	21,82	1,12	0,792				
Brainstem cm3 12mo	21,92	0,70	21,85	1,26	21,97	0,85	0,734	0,753	0,481	0,75	0,483
Brainstem cm3 24mo	21,66	0,70	21,58	0,98	21,73	1,03	1				
Brainstem % BL	1,58	0,04	1,58	0,09	1,57	0,05	0,624				
Brainstem % 12mo	1,57	0,04	1,55	0,08	1,59	0,04	0,238	0,333	0,719	0,312	0,735
Brainstem % 24mo	1,56	0,04	1,52	0,07	1,58	0,04	0,521				
LatVentricles cm3 BL	15,96	1,73	13,49	1,66	17,61	2,61	0,343				
**LatVentricles cm3 12mo**	**18,02**	**2,40**	**12,88**	**1,59**	**21,45**	**3,58**	**0,082**	0,104	0,901	0,114	0,79
LatVentricles cm3 24mo	17,55	2,34	13,05	0,99	20,54	3,66	0,27				
LatVentricles % BL	1,13	0,12	0,96	0,13	1,25	0,17	0,305				
**LatVentricles % 12mo**	**1,28**	**0,16**	**0,92**	**0,13**	**1,52**	**0,24**	**0,039**	0,097	0,908	0,104	0,902
LatVentricles % 24mo	1,25	0,16	0,92	0,07	1,46	0,25	0,238				
Caudate cm3 BL	5,53	0,31	5,59	0,46	5,49	0,43	0,851				
Caudate cm3 12mo	5,66	0,29	5,65	0,56	5,67	0,33	0,97	3,16	0,058	3,146	0,082
Caudate cm3 24mo	5,78	0,28	6,17	0,50	5,53	0,32	0,181				
Caudate % BL	0,40	0,03	0,40	0,04	0,40	0,04	0,678				
Caudate % 12mo	0,41	0,02	0,40	0,04	0,42	0,03	0,97	3,273	0,072	3,228	0,056
Caudate % 24mo	0,42	0,02	0,43	0,04	0,41	0,03	0,27				
Putamen cm3 BL	6,99	0,32	7,45	0,54	6,69	0,38	0,427				
Putamen cm3 12mo	6,98	0,30	7,54	0,51	6,62	0,35	0,157	2,758	0,081	2,586	0,095
Putamen cm3 24mo	7,20	0,28	7,75	0,53	6,84	0,29	0,157				
Putamen % BL	0,50	0,02	0,52	0,04	0,49	0,03	0,384				
Putamen % 12mo	0,50	0,02	0,53	0,03	0,48	0,03	0,238	2,718	0,083	2,537	0,099
Putamen % 24mo	0,52	0,02	0,54	0,03	0,50	0,03	0,343				
Thalamus cm3 BL	8,01	0,37	8,69	0,75	7,55	0,33	0,115				
Thalamus cm3 12mo	7,94	0,31	8,45	0,59	7,61	0,31	0,115	0,8	0,459	0,794	0,463
**Thalamus cm3 24mo**	**7,93**	**0,36**	**8,74**	**0,64**	**7,39**	**0,35**	**0,069**				
Thalamus % BL	0,58	0,03	0,61	0,05	0,55	0,03	0,305	0,677	0,516	0,676	0,517

Thalamus % 12mo	0,57	0,02	0,60	0,04	0,56	0,03	0,343				
**Thalamus % 24mo**	**0,57**	**0,02**	**0,61**	**0,04**	**0,54**	**0,03**	**0,098**				
GlobusPallidus cm3 BL	1,91	0,15	1,94	0,28	1,89	0,19	1				
GlobusPallidus cm3 12mo	1,86	0,11	1,79	0,23	1,90	0,10	0,384	2,849	0,075	2,665	0,089
GlobusPallidus cm3 24mo	1,91	0,11	1,93	0,18	1,89	0,14	1				
GlobusPallidus % BL	0,14	0,01	0,14	0,02	0,14	0,01	0,97				
GlobusPallidus % 12mo	0,13	0,01	0,13	0,01	0,14	0,01	0,238	2,979	0,067	2,802	0,079
GlobusPallidus % 24mo	0,14	0,01	0,13	0,01	0,14	0,01	0,851				
Hippocampus cm3 BL	7,82	0,37	7,65	0,63	7,93	0,47	0,792				
Hippocampus cm3 12mo	7,75	0,27	7,72	0,56	7,76	0,27	0,97	2,147	0,136	2,036	0,151
Hippocampus cm3 24mo	7,95	0,33	7,90	0,53	7,98	0,43	1				
Hippocampus % BL	0,56	0,03	0,54	0,05	0,58	0,03	0,851				
Hippocampus % 12mo	0,56	0,02	0,55	0,04	0,56	0,02	0,91	1,836	0,178	1,741	0,195
Hippocampus % 24mo	0,57	0,02	0,56	0,04	0,58	0,03	0,624				
Amygdala cm3 BL	1,53	0,12	1,51	0,20	1,55	0,17	0,734				
Amygdala cm3 12mo	1,49	0,11	1,49	0,24	1,49	0,10	0,734	**4,928**	**0,015**	**5,8**	**0,008**
Amygdala cm3 24mo	1,53	0,13	1,76	0,24	1,38	0,12	0,208				
Amygdala % BL	0,11	0,01	0,11	0,01	0,11	0,01	0,91				
Amygdala % 12mo	0,11	0,01	0,11	0,02	0,11	0,01	0,624	**5,42**	**0,01**	**6,099**	**0,007**
Amygdala % 24mo	0,11	0,01	0,12	0,02	0,10	0,01	0,343				
Accumbens cm3 BL	0,43	0,05	0,41	0,10	0,45	0,06	0,521				
Accumbens cm3 12mo	0,37	0,04	0,38	0,08	0,37	0,05	0,91	1,823	0,18	1,852	0,177
Accumbens cm3 24mo	0,41	0,04	0,45	0,08	0,38	0,05	0,384				
Accumbens % BL	0,03	0,00	0,03	0,01	0,03	0,01	0,473				
Accumbens % 12mo	0,03	0,00	0,03	0,01	0,03	0,00	0,678	1,107	0,326	1,26	0,293
Accumbens % 24mo	0,03	0,00	0,03	0,01	0,03	0,00	0,384				
ICV cm3 BL	1465,91	72,93	1436,48	42,17	1485,54	120,30	0,678				
ICV cm3 12mo	1394,97	28,94	1409,78	29,72	1385,09	44,88	0,91	0,483	0,501	0,431	0,526
ICV cm3 24mo	1409,93	29,82	1415,89	25,51	1405,61	49,28	1				
LesionCount BL	20,25	2,86	18,13	4,65	21,67	3,73	0,624				
LesionCount 12mo	19,40	2,62	20,50	5,63	18,67	2,50	0,792	0,292	0,641	0,348	0,603
LesionCount 24mo	20,60	2,67	20,75	5,72	20,50	2,58	0,521				
LesionVol cm3 BL	11,69	3,03	6,69	2,21	15,03	4,68	0,208	0,952	0,398	1,011	0,378

LesionVol cm3 12mo	9,60	2,71	9,29	4,67	9,81	3,44	0,624				
LesionVol cm3 24mo	10,34	2,72	10,27	4,73	10,38	3,44	0,624				
LesionVol (norm.) BL	0,71	0,15	0,46	0,16	0,86	0,22	0,268				
LesionVol (norm.) 12mo	0,66	0,18	0,64	0,31	0,67	0,23	0,571	1,646	0,211	1,703	0,202
LesionVol (norm.) 24mo	0,67	0,19	0,71	0,32	0,64	0,24	1				
LesionBurden BL	2,35	0,69	1,44	0,54	2,96	1,08	0,305				
LesionBurden 12mo	1,93	0,58	1,58	0,77	2,17	0,85	0,473	0,007	0,993	0,007	0,934
LesionBurden 24mo	1,99	0,58	1,68	0,78	2,20	0,85	0,571				
PeriventrLesionCount BL	5,14	0,58	4,13	0,44	5,77	0,86	0,185				
PeriventrLesionCount 12mo	5,05	0,73	3,63	0,71	6,00	1,06	0,135	0,147	0,723	0,137	0,732
PeriventrLesionCount 24mo	5,45	0,91	4,63	1,15	6,00	1,33	0,734				
PeriventrLesionVol cm3 BL	8,74	2,16	5,35	2,07	10,99	3,23	0,208				
PeriventrLesionVol cm3 12mo	8,49	2,55	7,87	4,13	8,91	3,40	0,571	0,965	0,353	0,953	0,357
PeriventrLesionVol cm3 24mo	8,54	2,61	8,82	4,23	8,34	3,47	0,851				
PeriventrLesionVol (norm.) BL	0,55	0,13	0,38	0,15	0,67	0,18	0,27				
PeriventrLesionVol (norm.) 12mo	0,58	0,17	0,55	0,28	0,61	0,23	0,521	1,302	0,279	1,256	0,088
PeriventrLesionVol (norm.) 24mo	0,59	0,18	0,62	0,29	0,58	0,23	0,91				
PeriventrLesionBurden BL	1,71	0,44	1,07	0,41	2,14	0,68	0,343				
PeriventrLesionBurden 12mo	1,71	0,56	1,33	0,68	1,97	0,83	0,473	0,075	0,827	0,068	0,835
PeriventrLesionBurden 24mo	1,57	0,56	1,43	0,69	1,67	0,83	0,792				
JuxtacLesionCount BL	11,00	2,27	11,50	4,41	10,67	2,57	0,851				
JuxtacLesionCount 12mo	9,90	2,42	12,50	5,23	8,17	2,15	0,624	0,74	0,486	0,774	0,471
JuxtacLesionCount 24mo	10,00	2,62	11,88	5,31	8,75	2,74	0,624				
JuxtacLesionVol cm3 BL	1,84	0,77	1,29	0,61	2,20	1,23	0,473				
JuxtacLesionVol cm3 12mo	0,98	0,28	1,29	0,61	0,77	0,24	0,571	0,376	0,557	0,418	0,535
JuxtacLesionVol cm3 24mo	1,07	0,34	1,33	0,66	0,90	0,38	0,624				
JuxtacLesionVol (norm.) BL	0,12	0,05	0,09	0,04	0,15	0,08	0,851				
JuxtacLesionVol (norm.) 12mo	0,07	0,02	0,09	0,04	0,05	0,02	0,624	0,414	0,537	0,459	0,517
JuxtacLesionVol (norm.) 24mo	0,07	0,02	0,09	0,04	0,06	0,03	0,521				
JuxtacLesionBurden BL	0,35	0,13	0,36	0,21	0,35	0,17	0,792				
JuxtacLesionBurden 12mo	0,19	0,05	0,23	0,10	0,17	0,06	0,91	0,066	0,811	0,058	0,823
JuxtacLesionBurden 24mo	0,19	0,06	0,23	0,12	0,15	0,07	0,521				
DeepWhiteLesionCount BL	4,05	0,82	2,38	0,75	5,17	1,18	0,208	0,226	0,779	0,222	0,72

DeepWhiteLesionCount 12mo	4,45	0,55	4,38	0,73	4,50	0,81	1				
DeepWhiteLesionCount 24mo	4,10	0,70	4,25	0,80	4,00	1,06	0,473				
DeepWhiteLesionVol cm3 BL	1,12	1,03	0,05	0,01	1,83	1,71	0,27				
DeepWhiteLesionVol cm3 12mo	0,16	0,04	0,22	0,09	0,12	0,03	0,734	0,227	0,642	0,198	0,664
DeepWhiteLesionVol cm3 24mo	0,10	0,03	0,12	0,05	0,08	0,04	0,427				
DeepWhiteLesionVol (norm.) BL	0,04	0,04	0,00	0,00	0,07	0,06	0,157				
DeepWhiteLesionVol (norm.) 12mo	0,01	0,00	0,01	0,01	0,01	0,00	0,773	0,198	0,664	0,183	0,677
DeepWhiteLesionVol (norm.) 24mo	0,01	0,00	0,01	0,00	0,01	0,00	0,384				
DeepWhiteLesionBurden BL	0,29	0,28	0,01	0,00	0,48	0,46	0,181				
DeepWhiteLesionBurden 12mo	0,02	0,01	0,02	0,01	0,03	0,01	0,473	0,221	0,646	0,193	0,667
DeepWhiteLesionBurden 24mo	0,02	0,01	0,02	0,01	0,01	0,01	0,427				
**CerebellumVol cm3 BL**	**130,27**	**4,23**	**139,97**	**7,98**	**123,80**	**3,89**	**0,082**				
**CerebellumVol cm3 12mo**	**126,86**	**4,32**	**136,94**	**7,88**	**120,15**	**4,13**	**0,057**	**3,684**	**0,065**	**3,729**	**0,065**
CerebellumVol cm3 24mo	124,91	4,64	129,69	10,34	121,73	3,79	0,208				
CerebellumVol % BL	9,30	0,22	9,76	0,43	8,99	0,19	0,069				
**CerebellumVol % 12mo**	**9,19**	**0,21**	**9,68**	**0,43**	**8,85**	**0,16**	**0,02**	**3,372**	**0,083**	**3,285**	**0,088**
CerebellumVol % 24mo	8,99	0,29	9,22	0,69	8,84	0,18	0,115				
**CerebellumGMVol cm3 BL**	**94,76**	**4,15**	**105,46**	**8,33**	**87,64**	**2,94**	**0,01**				
**CerebellumGMVol cm3 12mo**	**91,03**	**3,53**	**98,93**	**5,94**	**85,77**	**3,82**	**0,025**	**4,342**	**0,23**	**5,082**	**0,014**
CerebellumGMVol cm3 24mo	90,02	3,64	92,66	7,42	88,26	3,75	0,305				
**CerebellumGMVol % BL**	**6,76**	**0,24**	**7,34**	**0,49**	**6,36**	**0,17**	**0,02**				
**CerebellumGMVol % 12mo**	**6,54**	**0,19**	**7,00**	**0,34**	**6,23**	**0,19**	**0,02**	**4,279**	**0,024**	**4,862**	**0,016**
CerebellumGMVol % 24mo	6,48	0,24	6,59	0,50	6,41	0,23	0,27				
CerebellumCorticalThikness cm3 BL	2,35	0,27	2,39	0,39	2,32	0,38	0,521				
CerebellumCorticalThikness cm3 12mo	2,23	0,28	1,91	0,16	2,45	0,45	0,792	0,831	0,446	0,878	0,428
CerebellumCorticalThikness cm3 24mo	2,38	0,28	2,37	0,31	2,40	0,43	0,521				
CerebellumCorticalThikness (norm.) BL	2,09	0,24	2,11	0,34	2,08	0,35	0,473				
CerebellumCorticalThikness (norm.) 12mo	2,00	0,25	1,70	0,14	2,20	0,41	0,851	0,833	0,445	0,879	0,394
CerebellumCorticalThikness (norm.) 24mo	2,14	0,25	2,11	0,27	2,15	0,39	0,473				

SE: Standard Error of Mean; BL: Baseline; 12mo: 12 months; 24mo: 24 months; WM: White Matter; GM: Grey; Matter; CSF: Cerebro-spinal fluid; IC: Intra-cerebral; LatVentricles: Lateral ventricles; ICV: Intra-cerebral volume; LesionVol: Lesion volume; norm: normalized; *Mann-Whitney U test for responders vs. non-responders; bold indicates measurements with p<0.01.

Table S2. % mean reduction in cerebellar volume and in Grey Matter cerebellar volume from baseline at 12 – and at 24 months for responders at 24 months versus non-responders at 24 months

Parameter	Non-responders (N=8)		Responders (N=12)		p*
	% Mean change from baseline	SE	% Mean change from baseline	SE	
Cerebrum GM cm^3 12mo	18.54	9.49	20.95	31.55	0.663
Cerebrum GM cm^3 24mo	-4.38	25.17	7.08	25.47	0.58
Cerebrum GM % 12mo	1.68	0.68	1.86	2.17	0.399
Cerebrum GM % 24mo	-0.1	1.73	0.92	1.73	0.294
CerebellumVol cm^3 12mo	-2,14	1,47	-2,91	1,49	0.46
CerebellumVol cm^3 24mo	-6,38	6,63	-1,58	1,16	0.788
CerebellumVol % 12mo	-0,75	1,59	-1,35	0,98	0.718
CerebellumVol % 24mo	-4,98	6,66	-1,47	1,55	0.713
CerebellumGMVol cm^3 12mo	-4,93	3,44	-2,27	2,38	0.931
CerebellumGMVol cm^3 24mo	-10,35	6,52	0,82	2,94	0.681
CerebellumGMVol % 12mo	-3,70	3,16	-1,82	2,48	0.748
CerebellumGMVol % 24mo	-9,10	6,41	0,98	3,18	0.442
LesionCount 12mo	2.38	2.58	-3	3.28	0.872
LesionCount 24mo	2.63	2.99	-1.17	3.7	0.754
LesionVol cm^3 12mo	2.6	3.88	-5.22	3.8	0.4
LesionVol cm^3 24mo	3.58	3.71	-4.65	3.78	0.426
LesionVol (norm.) 12mo	0.18	0.27	-0.22	0.18	0.48
LesionVol (norm.) 24mo	0.25	0.26	-0.25	0.21	0.579
LesionBurden 12mo	0.14	0.33	-0.72	1.03	0.365
LesionBurden 24mo	0.24	0.32	-0.76	1.03	0.383

SE: Standard Error of Mean; BL: Baseline; 12mo: 12 months; 24mo: 24 months; GM: Grey; Matter; * Repeated Measures - General Linear Model mean comparison between responders and non-responders.

Table S3. Association of volumetry and lesion analysis parameters' variability with cognitive and EDSS scores' variability over time (only associations with p values <0.1 are presented).

Dependent variable	Fixed factor (in addition to time)	-2 Restricted Log Likelihood	Estimate	p
**SDMT**	**LatVentricles_abs**	**364.219**	**-0.34**	**0.015**
	**LatVentricles_percent**	**358.689**	**-4.97**	**0.012**
	Hippocampus_abs	364.554	-0.86	0.093
	Hippocampus_percent	358.921	-13.17	0.078
	**Amygdala_percent**	**356.453**	**-39.33**	**0.047**
	**ICV_abs**	**369.751**	**0.008**	**0.018**
	**LesionCount**	**362.004**	**0.26**	**0.001**
	**PeriventrLesionCount**	**371.799**	**0.57**	**0.004**
	JuxtacLesionCount	369.433	0.19	0.05
	**DeepWhiteLesionVol_abs**	**367.436**	**0.41**	**0.035**
	**DeepWhiteLesionVol_norm**	**356.802**	**11.09**	**0.042**
	**DeepWhiteLesionBurden**	**364.667**	**1.57**	**0.033**
	CerebellumVol_abs	368.819	0.08	0.068
	**CerebellumGMVol_abs**	**365.829**	**0.12**	**0.012**
	**CerebellumGMVol_percent**	**361.441**	**1.75**	**0.022**
**GVLT**	**LatVentricles_abs**	**416.139**	**-0.47**	**0.012**
	**LatVentricles_percent**	**411.061**	**-6.71**	**0.012**
	**Thalamus_abs**	**415.626**	**1.99**	**0.047**
	**Thalamus_percent**	**409.107**	**30.34**	**0.03**
	DeepWhiteLesionVol_abs	420.862	0.74	0.052
	DeepWhiteLesionVol_norm	408.786	20.27	0.057
	**DeepWhiteLesionBurden**	**418.092**	**2.84**	**0.049**
	CerebellumCorticalThikness_abs	414.720	-2.4	0.059
	CerebellumCorticalThikness_norm	414.448	-2.73	0.057
**BVLT-R**	**LatVentricles_abs**	**394.187**	**-0.35**	**0.02**
	**LatVentricles_percent**	**387.626**	**-5.57**	**0.009**

	**Putamen_abs**	**391.533**	**1.94**	**0.027**
	Putamen_percent	387.9	20.88	0.094
	**Thalamus_abs**	**391.356**	**1.86**	**0.026**
**MFIS**	WM_abs	481.15	0.04	0.092
	**IC_abs**	**479.579**	**0.05**	**0.045**
	**LatVentricles_abs**	**468.472**	**0.97**	**<0.001**
	**LatVentricles_percent**	**464.94**	**3.9**	**0.002**
	**Thalamus_percent**	**465.154**	**-50.71**	**0.018**
	**JuxtacLesionVol_abs**	**467.674**	**1.57**	**0.034**
	**JuxtacLesionVol_norm**	**462.549**	**22.42**	**0.04**
	**JuxtacLesionBurden**	**464.342**	**10.58**	**0.031**
	**CerebellumVol_percent**	**468.597**	**-5.66**	**0.008**
	CerebellumGMVol_abs	477.778	-0.23	0.098
	**CerebellumGMVol_percent**	**468.794**	**-5.52**	**0.013**
**EDSS**	CSF_percent	147.898	-0.03	0.068
	Brain_abs	152.772	0.002	0.059
	Brain_percent	147.898	-0.03	0.068
	Cerebrum_abs	152.540	0.002	0.053
	Cerebrum_percent	147.585	0.03	0.059
	Cerebelum_WM_abs	148.089	0.02	0.051
	Cerebelum_WM_percent	143.482	0.24	0.08

SDMT, Symbol Digit Modalities Test; GVLT, Greek Verbal Learning Test; BVMT-R, Brief Visuospatial Memory Test-Revised; MFIS, Modified Fatigue Impact Scale; EDSS: Expanded Disability Status Scale. Bold indicates significant associations between volumetry and/or lesion analysis parameters' variability with cognitive and/or EDSS scores' variability over time.

Table S4. Immune cell phenotype assessed at baseline pre-1st infusion (BLa) and 15 days after treatment with the 1st infusion and pre-2nd infusion (BLb), as well as in 6-month intervals thereafter, prior to the next scheduled infusion of ocrelizumab and % change from baseline for 6-month interval point estimates for all patients and non-responders at 24 months versus responders at 24 months.

	absolute cell counts or %										% mean change from BLa									
	All patients			Non-responders			Responders				All patients			Non-responders			Responders			
Cell subset	N	Mean	SE	N	Mean	SE	N	Mean	SE	p*	N	Mean	SE	N	Mean	SE	N	Mean	SE	p*
#WBCs BLa	21	7,48	0,32	9	7,11	0,46	12	7,75	0,43	0,247										
#WBCs BLb	21	7,35	0,47	9	7,33	0,58	12	7,36	0,73	0,917	21	-1,89	4,37	9	3,79	5,56	12	-6,15	6,34	0,193
#WBCs 6mo	21	6,73	0,38	9	6,59	0,79	12	6,83	0,34	0,651	21	-9,40	4,25	9	-8,69	7,33	12	-9,93	5,29	1
#WBCs 12mo	21	7,76	0,65	9	6,91	0,54	12	8,39	1,04	0,193	21	3,59	6,35	9	-1,33	8,11	12	7,28	9,47	0,554
#WBCs 18mo	19	7,16	0,55	8	7,59	1,17	11	6,85	0,48	0,968	19	-1,76	6,50	8	7,44	13,34	11	-8,45	5,47	0,6
#WBCs 24mo	16	7,83	0,83	6	8,60	1,91	10	7,36	0,75	0,875	16	0,93	7,49	6	8,08	17,82	10	-3,36	6,18	0,958
#lymp/tes BLa	22	1894,55	84,97	**9**	**1647,78**	**110,25**	**13**	**2065,38**	**99,12**	**0,011**										
#lymp/tes BLb	21	1510,48	72,80	8	1410,00	63,78	13	1572,31	109,41	0,336	21	-20,42	3,34	8	-16,91	3,54	13	-22,58	4,96	0,301
#lymp/tes 6mo	22	1463,64	83,91	**9**	**1316,67**	**129,91**	**13**	**1565,38**	**104,65**	**0,071**	22	-20,58	4,54	9	-17,75	8,30	13	-22,55	5,34	0,794
#lymp/tes 12mo	21	1653,81	140,60	8	1495,00	152,68	13	1751,54	206,79	0,547	21	-13,52	4,53	8	-10,02	4,84	13	-15,67	6,76	0,456
#lymp/tes 18mo	19	1587,37	102,79	8	1601,25	170,59	11	1577,27	134,19	0,968	19	-12,64	6,28	8	-1,26	10,55	11	-20,91	7,06	0,177
#lymp/tes 24mo	16	1487,50	91,05	6	1571,67	144,44	10	1437,00	119,98	0,428	16	-16,93	6,62	6	2,46	10,24	10	-28,57	6,46	0,031
#CD19 BLa	22	102,37	9,22	9	100,21	15,47	13	103,87	11,87	0,647										
#CD19 BLb	21	0,60	0,09	8	0,66	0,16	13	0,56	0,12	0,595	21	-99,23	0,20	8	-98,94	0,49	13	-99,40	0,13	0,697
#CD19 6mo	22	8,63	3,13	9	8,54	5,15	13	8,70	4,09	0,948	22	-92,88	1,80	9	-92,84	2,78	13	-92,90	2,46	0,896
#CD19 12mo	21	4,00	1,40	8	5,68	2,69	13	2,97	1,57	0,301	21	-96,07	1,20	8	-94,88	2,14	13	-96,80	1,45	0,336
#CD19 18mo	19	1,81	0,69	8	2,39	1,48	11	1,40	0,58	0,968	16	-91,58	3,04	6	-85,66	6,75	10	-95,14	2,37	0,428
#CD19 24mo	16	8,42	4,26	6	15,64	10,96	10	4,10	1,67	0,635	16	-91,58	3,04	6	-85,66	6,75	10	-95,14	2,37	0,428
%CD19 BLa	22	9,84	0,89	9	9,63	1,49	13	9,99	1,14	0,647										
%CD19 BLb	21	0,06	0,01	8	0,06	0,01	13	0,05	0,01	0,645	21	-99,23	0,20	8	-98,97	0,47	13	-99,39	0,14	0,75
%CD19 6mo	22	0,83	0,30	9	0,82	0,49	13	0,84	0,39	1	22	-92,89	1,80	9	-92,87	2,77	13	-92,91	2,47	0,794
%CD19 12mo	22	0,39	0,13	9	0,49	0,23	13	0,33	0,15	0,695	22	-95,97	1,13	9	-95,39	1,95	13	-96,38	1,40	0,647
%CD19 18mo	21	0,33	0,22	9	0,08	0,03	12	0,52	0,38	0,31	21	-97,13	1,33	9	-98,81	0,48	12	-95,86	2,27	0,345
%CD19 24mo	16	0,59	0,31	6	1,08	0,79	10	0,29	0,12	0,635	16	-94,36	2,00	6	-90,40	4,62	10	-96,73	1,35	0,492
%pl/blasts BLa	22	1,43	0,31	9	1,61	0,60	13	1,30	0,34	0,794										

%pl/blasts BLb	21	19,14	6,31	8	23,06	11,40	13	16,73	7,70	0,804	18	1428,39	619,06	7	1586,93	1144,15	11	1327,50	750,75	0,93
%pl/blasts 6mo	22	13,82	4,15	**9**	**22,66**	**7,19**	**13**	**7,70**	**4,40**	**0,043**	19	1419,01	607,94	8	2755,10	1294,52	11	447,32	267,80	0,109
%pl/blasts 12mo	21	25,62	6,04	**8**	**39,54**	**10,62**	**13**	**17,05**	**6,46**	**0,076**	18	2218,15	967,75	7	3760,24	2300,94	11	1236,81	579,80	0,246
%pl/blasts18mo	20	21,56	5,02	8	18,57	8,45	12	23,56	6,43	0,624	17	1505,01	655,39	7	394,37	303,91	10	2282,45	1044,97	0,193
%pl/blasts 24mo	16	22,28	6,97	6	9,98	4,23	10	29,66	10,37	0,368	13	2871,89	1843,25	5	333,96	132,83	8	4458,10	2919,56	0,171
%tr. CD19+ BLa	19	2,33	1,32	9	1,47	0,80	10	3,11	2,45	1										
%tr. CD19+ BLb	18	0,00	0,00	8	0,00	0,00	10	0,00	0,00	1	11	-100,00	0,00	5	-100,00	0,00	6	-100,00	0,00	1
%tr. CD19+ 6mo	19	14,65	4,06	9	9,82	5,32	10	19,01	5,95	0,278	12	1781,30	863,03	**6**	**349,25**	**205,99**	**6**	**3213,35**	**1553,87**	**0,026**
%tr. CD19+12mo	18	7,22	3,04	8	6,28	3,94	10	7,96	4,64	0,897	12	986,26	537,54	6	389,71	351,84	6	1582,81	1002,60	0,485
%tr. CD19+ 18mo	17	6,57	4,05	8	0,00	0,00	9	12,40	7,28	0,139	12	307,53	386,10	6	-100,00	0,00	6	715,05	767,78	0,394
%tr. CD19+ 24mo	13	15,87	6,50	6	11,95	8,00	7	19,22	10,34	0,731	11	3010,12	1644,57	5	2945,92	2953,12	6	3063,62	2025,26	0,792
%MZ BLa	22	16,45	2,68	9	14,02	2,97	13	18,12	4,08	0,948										
%MZ BLb	21	0,00	0,00	8	0,00	0,00	13	0,00	0,00	1	20	-100,00	0,00	7	-100,00	0,00	13	-100,00	0,00	1
%MZ 6mo	22	1,09	0,46	9	1,29	0,77	13	0,95	0,60	0,744	21	-90,53	4,76	8	-91,08	6,74	13	-90,19	6,68	0,972
%MZ 12mo	21	0,53	0,30	8	0,90	0,61	13	0,31	0,31	0,547	20	-90,62	7,60	7	-76,27	21,43	13	-98,34	1,66	0,485
%MZ 18mo	20	0,98	0,81	8	0,00	0,00	12	1,63	1,34	0,384	19	-94,18	4,60	7	-100,00	0,00	12	-90,78	7,22	0,384
%MZ 24mo	16	0,41	0,28	6	0,59	0,59	10	0,29	0,29	0,792	15	-90,48	8,20	5	-75,45	24,55	10	-97,99	2,01	0,768
%cl. sw.BLa	22	12,58	1,47	9	12,03	2,01	13	12,96	2,12	0,948										
%cl. sw.BLb	21	2,50	1,77	8	4,07	4,07	13	1,53	1,53	0,86	20	-86,11	10,06	7	-73,99	26,01	13	-92,64	7,36	0,817
%cl. sw.6mo	22	10,65	2,62	9	12,28	4,63	13	9,52	3,19	0,744	21	-26,35	18,63	8	-19,61	29,66	13	-30,49	24,79	0,697
%cl. sw.12mo	21	9,64	3,40	8	8,44	5,66	13	10,37	4,42	0,972	18	-7,44	1,41	8	-5,57	2,09	10	-8,94	1,86	0,408
%cl. sw.18mo	20	8,79	3,16	8	7,27	4,98	12	9,81	4,24	0,624	19	-48,96	18,39	7	-46,80	34,62	12	-50,22	22,24	0,837
%cl. sw.24mo	16	6,63	2,08	6	8,51	3,81	10	5,50	2,51	0,562	15	-57,63	13,05	5	-28,98	23,65	10	-71,95	14,30	0,165
%non-sw. BLa	19	1,00	0,15	9	0,82	0,19	10	1,16	0,24	0,604										
%non-sw. BLb	19	0,00	0,00	9	0,00	0,00	10	0,00	0,00	1	18	-100,00	0,00	8	-100,00	0,00	10	-100,00	0,00	1
%non-sw. 6mo	19	0,00	0,00	9	0,00	0,00	10	0,00	0,00	1	18	-100,00	0,00	8	-100,00	0,00	10	-100,00	0,00	1
%non-sw. 12mo	19	0,11	0,11	9	0,24	0,24	10	0,00	0,00	0,72	18	-89,19	10,81	8	-75,68	24,32	10	-100,00	0,00	0,696
%non-sw. 18mo	16	1,05	1,05	8	0,00	0,00	8	2,11	2,11	0,721	15	72,82	172,82	7	-100,00	0,00	8	224,04	324,04	0,694
%non-sw. 24mo	13	0,30	0,23	6	0,65	0,48	7	0,00	0,00	0,366	12	-53,84	34,61	5	10,79	78,21	7	-100,00	0,00	0,268
%naive B BLa	22	58,64	4,24	9	54,91	7,65	13	61,22	4,98	0,601										
%naive B BLb	22	0,00	0,00	9	0,00	0,00	13	0,00	0,00	1	21	-100,00	0,00	8	-100,00	0,00	13	-100,00	0,00	1

%naive B 6mo	22	36,70	7,43	9	31,52	11,68	13	40,28	9,90	0,744	21	-43,21	12,09	8	-57,75	18,17	13	-34,26	16,04	0,374
%naive B 12mo	21	16,66	5,70	8	19,51	10,05	13	14,91	7,12	0,75	20	-72,59	10,49	7	-72,95	17,79	13	-72,40	13,51	0,938
%naive B 18mo	20	16,10	6,95	8	8,17	8,17	12	21,40	10,22	0,305	19	-72,37	11,53	7	-82,20	17,80	12	-66,63	15,34	0,432
%naive B 24mo	16	28,27	7,95	6	39,79	14,14	10	21,36	9,37	0,313	15	-47,98	14,61	5	-27,39	21,70	10	-58,28	18,92	0,44
#CD3+ BLa	22	754,71	15,70	**9**	**713,81**	**15,10**	**13**	**783,03**	**21,48**	**0,043**										
#CD3+ BLb	19	825,90	15,76	**7**	**768,74**	**12,78**	**12**	**859,25**	**17,81**	**0,004**	19	8,59	2,11	7	5,82	2,20	12	10,21	3,06	0,482
#CD3+ 6mo	21	816,20	20,80	**9**	**741,45**	**26,36**	**12**	**872,26**	**18,21**	**0,001**	21	7,75	2,26	9	4,12	3,84	12	10,47	2,58	0,169
#CD3+ 12mo	15	813,69	30,07	7	772,72	45,86	8	849,55	37,68	0,281	15	7,58	3,03	7	9,66	5,46	8	5,75	3,33	0,397
#CD3+ 18mo	15	810,33	25,73	**5**	**717,88**	**45,14**	**10**	**856,56**	**19,48**	**0,008**	15	5,59	2,38	5	3,45	6,04	10	6,67	2,17	0,371
#CD3+ 24mo	13	1113,85	94,32	5	1029,86	101,19	8	1166,34	141,77	0,622	13	46,28	10,78	5	49,38	15,74	8	44,35	15,28	1
#Neutr/ls BLa	22	4618,18	283,20	9	4644,44	463,41	13	4600,00	371,93	0,896										
#Neutr/ls BLb	19	4831,58	434,48	7	4657,14	405,24	12	4933,33	659,01	0,837	19	5,47	5,40	7	6,29	8,13	12	4,99	7,38	0,902
#Neutr/ls mo	21	4671,43	512,61	9	5111,11	1164,69	12	4341,67	270,37	0,754	21	2,53	7,89	9	5,34	17,25	12	0,42	5,89	0,554
#Neutr/ls 12mo	19	5242,11	542,70	9	4611,11	423,43	10	5810,00	948,74	0,604	19	12,98	9,81	9	5,98	14,42	10	19,28	13,78	0,4
#Neutr/ls 18mo	17	4217,65	297,44	5	3900,00	727,32	12	4350,00	311,03	0,383	17	-3,33	7,62	5	-11,62	22,54	12	0,12	6,24	0,279
#Neutr/ls 24mo	13	5130,77	571,37	5	4720,00	513,23	8	5387,50	889,31	0,833	13	5,72	6,26	5	-3,06	6,68	8	11,21	9,06	0,435

#: absolute cell counts; WBCs: white blood cells (K/μl) ;lymp/tes: lymphocytes/μl; CD19: CD19+ cells/μl; pl/blasts: plasmablasts (% of CD19+ cells); tr. CD19: transitional B-cells (% of CD19+ cells); MZ: Marginal zone-like B-cells (% of CD19+ cells); cl.sw.: class-switched B-cells (% of CD19+ cells); non-sw.: non-switched B-cells (% of CD19+ cells); naive B: naive B-cells (% of CD19+ cells); CD3: CD3+ cells/μl; Neutr/ls: neurtrophils (cells/μl); BLa: baseline pre-1st infusion; BLb: 15 days after treatment with the 1st infusion and pre-2nd infusion; SE: Standard Error of Mean; 6mo: 6 months; 12mo: 12 months; 18mo: 18 months; 24mo: 24 months; *Mann-Whitney U test for non-responders at 24 months versus responders at 24 months; bold indicates measurements with p<0.01.

Table S5. Cytokine levels assessed at baseline pre-1st infusion (BL), as well as in 6- and 12-month time-points, prior to the next scheduled infusion of ocrelizumab and % mean cytokine change from baseline for 6- and 12-month time-points for all patients and non-responders at 24 months versus responders at 24 months.

	pg/ml										% mean change from BL									
	All patients			Non-responders			Responders				All patients			Non-responders			Responders			
Cytokine	N	Mean	SE	N	Mean	SE	N	Mean	SE	p	N	Mean	SE	N	Mean	SE	N	Mean	SE	p
**IL-4 BL**	17	52,18	4,29	7	54,86	6,93	10	50,30	5,68	0,813										
**IL-5 BL**	17	148,06	18,35	7	180,86	33,18	10	125,10	19,03	0,23										
**IL-17A BL**	17	114,53	11,92	7	131,29	17,44	10	102,80	15,83	0,23										
**IFNγ BL**	17	175,47	18,19	7	201,00	34,87	10	157,60	18,62	0,601										
**IL-10 BL**	17	64,35	8,71	7	58,29	6,48	10	68,60	14,32	0,962										
**IL-12 BL**	17	1,76	0,55	7	2,29	1,29	10	1,40	0,31	1										
**IL-1b_BL**	17	37,41	3,94	7	38,14	3,45	10	36,90	6,43	0,669										
**IL-2 BL**	17	51,12	3,96	7	54,57	5,74	10	48,70	5,52	0,475										
**IL-6 BL**	17	109,59	11,85	7	132,86	18,16	10	93,30	14,10	0,161										
**TNFα BL**	17	81,12	7,95	7	89,86	14,51	10	75,00	9,10	0,536										
**IL-4 6mo**	20	46,10	4,15	9	47,78	6,05	11	44,73	5,91	0,603	17	8,99	9,19	7	17,29	13,37	10	3,18	12,76	0,475
**IL-5 6mo**	20	136,90	17,15	9	149,78	24,32	11	126,36	24,58	0,552	17	-8,59	19,43	7	17,08	17,68	10	-26,56	30,14	0,475
**IL-17A 6mo**	20	105,15	11,01	9	117,44	17,22	11	95,09	14,21	0,331	17	-6,60	19,94	7	15,91	14,13	10	-22,36	32,27	0,417
**IFNγ 6mo**	20	259,55	98,92	9	401,56	215,67	11	143,36	18,99	0,152	17	-46,35	51,48	7	-117,19	124,65	10	3,23	9,76	0,74
**IL-10 6mo**	20	54,30	4,51	9	58,67	6,37	11	50,73	6,39	0,295	17	7,12	8,13	7	0,45	14,60	10	11,79	9,72	0,475
**IL-12 6mo**	20	1,25	0,25	9	1,56	0,56	11	1,00	0,00	0,71	17	9,71	5,46	7	5,71	5,71	10	12,50	8,54	0,813
**IL-1b 6mo**	20	31,20	3,48	9	34,78	4,84	11	28,27	4,95	0,23	17	10,58	11,11	7	16,15	16,50	10	6,69	15,56	0,813
**IL-2 6mo**	20	47,45	3,58	9	53,11	5,11	11	42,82	4,75	0,152	17	3,37	7,19	7	3,07	12,24	10	3,58	9,29	0,813
**IL-6 6mo**	20	105,20	11,47	9	112,56	18,09	11	99,18	15,22	0,656	17	-31,35	38,76	7	20,24	12,58	10	-67,47	64,14	0,23
**TNFα 6mo**	20	73,15	8,53	9	78,44	11,24	11	68,82	12,83	0,456	17	12,73	6,69	7	19,66	8,34	10	7,87	9,82	0,475
**IL-4 12mo**	20	46,30	3,47	9	45,22	5,49	11	47,18	4,66	0,941	17	1,87	15,99	7	22,49	12,67	10	-12,56	25,33	0,315
**IL-5 12mo**	20	146,15	15,01	9	142,56	26,65	11	149,09	17,64	0,882	17	-35,96	42,79	7	25,75	15,74	10	-79,16	70,10	0,109
**IL-7A12mo**	20	108,70	10,36	9	110,00	11,24	11	107,64	16,97	0,941	17	-35,32	39,01	7	6,52	25,53	10	-64,60	63,75	0,417
**IFNγ 12mo**	20	162,15	12,01	9	161,78	19,36	11	162,45	15,89	0,941	17	-2,13	15,93	7	20,49	12,45	10	-17,97	25,03	0,315
**IL-10 12mo**	20	54,35	4,19	9	53,22	6,50	11	55,27	5,72	0,941	17	6,97	11,91	7	16,19	10,72	10	0,52	19,07	0,669

**IL-12 12mo**	20	1,05	0,05	9	1,00	0,00	11	1,09	0,09	0,766	17	12,65	7,05	7	12,86	12,86	10	12,50	8,54	1
**IL-1b 12mo**	20	33,65	3,79	9	31,33	5,47	11	35,55	5,40	0,656	17	-5,28	22,45	7	26,64	13,73	10	-27,61	36,07	0,475
**IL-2 12mo**	20	48,75	2,89	9	47,00	3,70	11	50,18	4,41	0,552	17	-0,87	11,76	7	15,68	9,52	10	-12,45	18,43	0,315
**IL-6 12mo**	20	100,40	8,87	9	96,78	12,11	11	103,36	13,17	0,503	17	-63,97	80,46	**7**	**31,41**	**10,08**	**10**	**-130,74**	**135,33**	**0,055**
**TNFα 12mo**	20	78,65	6,62	9	78,89	10,73	11	78,45	8,73	0,941	17	-11,03	23,05	7	18,56	11,52	10	-31,74	37,79	0,364

SE: Standard Error of Mean; BL: Baseline; 6mo: 6 months; 12mo: 12 months; IL: interleukin; IFNγ: interferon-γ; TNFα: Tumor Necrosis Factor-α; *Mann-Whitney U test for non-responders at 24 months versus responders at 24 months; bold indicates measurements with $p<0.01$.
